# KAT6A Condensates Impair PARP1 Trapping of PARP Inhibitors in Ovarian Cancer

**DOI:** 10.1002/advs.202400140

**Published:** 2024-07-08

**Authors:** Zhiyan Zhan, Jiarong Zhang, Huisheng Liang, Chong Wang, Li Hong, Wenxue Liu

**Affiliations:** ^1^ Department of Clinical Nutrition, Shanghai Children's Medical Center, School of Medicine Shanghai Jiao Tong University Shanghai 200127 China; ^2^ Clinical Research Center, Shanghai Children's Medical Center, School of Medicine Shanghai Jiao Tong University Shanghai 200127 China; ^3^ Department of Obstetrics and Gynecology Zhongshan Hospital Fudan University Shanghai 200032 China; ^4^ Department of Gynecology Zhongshan Hospital, Fudan University (Xiamen Branch) Xiamen 361000 China; ^5^ Department of Obstetrics and Gynecology, Shanghai General Hospital, School of Medicine Shanghai Jiao Tong University 85 Wujin Road Shanghai 200080 China

**Keywords:** APEX1, KAT6A, liquid‐liquid phase separation, ovarian cancer, PARP1 trapping

## Abstract

Most clinical PARP inhibitors (PARPis) trap PARP1 in a chromatin‐bound state, leading to PARPi‐mediated cytotoxicity. PARPi resistance impedes the treatment of ovarian cancer in clinical practice. However, the mechanism by which cancer cells overcome PARP1 trapping to develop PARPi resistance remains unclear. Here, it is shown that high levels of KAT6A promote PARPi resistance in ovarian cancer, regardless of its catalytic activity. Mechanistically, the liquid‐liquid phase separation (LLPS) of KAT6A, facilitated by APEX1, inhibits the cytotoxic effects of PARP1 trapping during PARPi treatment. The stable KAT6A‐PARP1‐APEX1 complex reduces the amount of PARP1 trapped at the DNA break sites. In addition, inhibition of KAT6A LLPS, rather than its catalytic activity, impairs DNA damage repair and restores PARPi sensitivity in ovarian cancer both in vivo and in vitro. In conclusion, the findings demonstrate the role of KAT6A LLPS in fostering PARPi resistance and suggest that repressing KAT6A LLPS can be a potential therapeutic strategy for PARPi‐resistant ovarian cancer.

## Graphical abstract

KAT6A condensates, facilitated by APEX1, inhibits the cytotoxic effects of PARP1 trapping during PARPi treatment. The stable KAT6A‐PARP1‐APEX1 complex reduces the amount of PARP1 trapped at DNA break sites, inducing PARPi resistance of ovarian cancer. Targeting KAT6A LLPS can be a potential therapeutic strategy for PARPi‐resistant ovarian cancer.

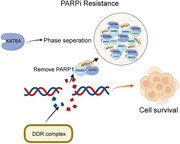

## 1 Introduction

Poly(ADP‐ribose)‐polymerase (PARP) inhibitors (PARPis) have gained approval for treating homologous recombination (HR)‐defective ovarian cancers.^[^ ^1^ ^]^ It has been reported that not only patients with BRCA1/2 mutations or HR deficiency^[^ ^2^ ^]^ benefit from PARPi treatment, but also those with platinum‐sensitive ovarian cancers.^[^ ^3^ ^]^ This benefit can be attributed to PARP1 trapping‐mediated cytotoxicity.^[^ ^3^ ^]^ However, a significant proportion of ovarian cancer cases result in recurrence, distant metastases, and acquired PARPi resistance. Therefore, it is crucial, irrespective of BRCA status, to better understand PARPi functions and resistance mechanisms, predict the clinical efficacy of PARPi treatment, and explore combination therapy options to improve the overall survival of patients with ovarian cancer.

Most clinical PARPis inhibit the catalytic activity of PARPs by binding to their NAD^+^ binding site (the catalytic domain), hindering DNA repair initiation. Additionally, most clinical PARPis trap PARP1 in a chromatin‐bound state, which drives PARPi‐mediated cytotoxicity.^[^ ^4^ ^]^ PARP1 deletion and mutations that impair PARP1 trapping can cause PARPi resistance.^[^ ^5^ ^]^ Tumors can escape the antitumor effects of PARPi by impairing PARP1 trapping. However, it remains unclear how PARP1 trapping becomes impaired in cancer cells.

We previously discovered that lysine acetyltransferase 6A (KAT6A, also known as MYST3 or MOZ) is overexpressed in ovarian cancer. It promotes tumorigenesis and platinum resistance through its catalytic function as an acetyltransferase.^[^ ^6^ ^]^ Additionally, KAT6A promotes the development of various cancers via its catalytic functions,^[^ ^7^ ^]^ while its non‐catalytic functions remain unclear.

In this study, we explored the relationship between KAT6A and PARPi resistance in ovarian cancer cells. We observed that deletion of KAT6A in PARP‐resistant ovarian cancer cells restored their sensitivity to PARPi. Mechanistically, we found that KAT6A undergoes LLPS in PARPi‐resistant cells, dependent on APEX1, thereby releasing trapped PARP1 from chromatin. Moreover, targeting KAT6A LLPS restored PARPi sensitivity, suggesting a promising clinical treatment strategy for PARPi‐resistant ovarian cancer.

## 2 Results

### 2.1 KAT6A Promotes PARPi Resistance in Ovarian Cancer Cells through its Noncatalytic Function

**Figure 1:**
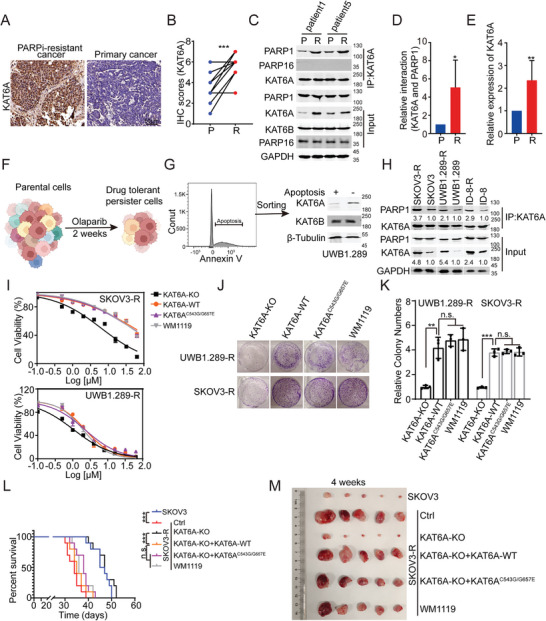
KAT6A promotes PARPi resistance of ovarian cancer cells through its non‐catalytic function. A,B) Both primary and PARPi‐resistant tumor tissues from 7 ovarian cancer patients were collected, and then the expression of KAT6A was analyzed by IHC assays. IHC assays showed that the expression of KAT6A is higher in PARPi‐resistant ovarian cancer cells (R) than that in primary cancer cells (P). The representative images are shown in A, and IHC staining scores of KAT6A are in B (Paired *t*‐test, *n* = 7). C–E) Co‐IP and Western blot assays showed that higher level of KAT6A and more interaction of KAT6A and PARP1 were detected in PARPi‐resistant ovarian cancer cells. PARP16 was set as negative control for Co‐IP assays. Quantification of C is shown in D and E (Paired t‐test, *n* = 7). F) Schematic representation of the establishment of PARPi‐resistant ovarian cancer cells (drug tolerant persister cells) using cell lines. The image was created by biorender.com. G) Apoptotic or non‐apoptotic UWB1.289 cells were sorted using Annexin V antibody during PARPi treatment (left), the expression of KAT6A but not KAT6B is higher in non‐apoptotic UWB1.289 cells than that in apoptotic UWB1.289 cells (right). Representative results from one of three independent experiments are shown. H) Co‐IP and Western blot assays showed that higher level of KAT6A and more interaction of KAT6A and PARP1 were detected in PARPi‐resistant ovarian cancer cells than that in primary cancer cells in vitro. I) UWB1.289‐R and SKOV3‐R cells of indicated genotypes were treated with olaparib at increasing concentrations for 72 h, after which cell viability was detected by Cell Titer‐Glo luminescent assay (replicates, *n* = 3; symbols represent averages of experimental replicates; error bars indicate SEM). The influence of WM‐1119 (25 µm) on cell‐inhibitory effects of olaparib was also assessed. J,K) KAT6A knockout inhibits colony formation of UWB1.289‐R and SKOV3‐R cells, whereas repressing catalytic function of KAT6A using KAT‐deficient mutants (KAT6A^C543G/G657E^) or WM1119 has no effects on colony formation of UWB1.289‐R and SKOV3‐R cells (one‐way ANOVA followed by Tukey's multiple‐comparison test, *n* = 3 per group). Representative images are shown in J and quantification is shown in K. L) Survival of mice injected i.p. with SKOV3 or SKOV3‐R cells of indicated genotypes. Mice were treated using PARPi (olaparib, 50 mg kg^−1^ d^−1^) and cisplatin (5 mg kg^−1^·d^−1^) during week 2–4. SKOV3‐R shortens the survival of mice compared to SKOV3. KAT6A‐KO prolongs the survival of mice, but KAT6A^C543G/G657E^ or WM1119 has no influence on the survival of mice (Log‐rank test, *n* = 10 per group). M) SKOV3 or SKOV3‐R cells of indicated genotypes were injected subcutaneously into the hind flanks of nu/nu mice. Mice were treated in the same way as shown in L. The representative image of tumor 4 weeks post injection is shown. Data are expressed as mean ± SD for D, E, K. *P < 0.05; **P < 0.01; ***P < 0.001; n.s. denotes no signification.

To investigate the role of KAT6A in PARPi resistance in ovarian cancer, we collected both primary and PARPi‐resistant tumor tissues from seven patients with ovarian cancer (patients 1–4 with BRCA deficiency were treated with olaparib, and patients 5–7 without BRCA deficiency were treated with niraparib according to the National Comprehensive Cancer Network [NCCN] guidelines, nccn.org/patients). The expression of KAT6A was higher in PARPi‐resistant ovarian tumors compared to primary ovarian cancer (**Figure** **1**A–D). Notably, our analysis of these clinical samples revealed more interactions between KAT6A and PARP1 in PARPi‐resistant ovarian tumors than in primary ovarian cancer (Figure 1C,E), suggesting a potential role of KAT6A in PARPi resistance.

To validate promotion of PARPi resistance by KAT6A, we used UWB1.289 (with HR deficiency), SKOV3 (without HR deficiency), and ID8 cells in our study.^[^ ^8^ ^]^ Next, we generated PARPi‐resistant ovarian cancer cell lines (SKOV3‐R, UWB1.289‐R, and ID‐8‐R) by exposing them to a lethal dose of a PARPi (olaparib) for 2 weeks^[^ ^9^ ^]^ (Figure 1F). Notably, compared to the parental cells, drug‐tolerant persister (DTP) cells exhibited significantly reduced sensitivity to PARPi both in vivo and in vitro (Figure S1A,B, Supporting Information). Annexin V staining was used to detect apoptotic cells during the generation of PARPi‐resistant ovarian cancer cells, and the KAT6A level in non‐apoptotic (DTP) cells was much higher than that in apoptotic cells (Figure 1G), indicating that ovarian cancer cells with high KAT6A levels survived PARPi treatment. Further analysis identified an interaction between KAT6A and PARP1 in ovarian cancer cells (Figure 1H), and this interaction between KAT6A and PARP1 was confirmed in HEK293T cells (Figure S1C, Supporting Information). Additionally, the interaction between KAT6A and PARP1 was enhanced in PARPi‐resistant ovarian cancer cells treated with olaparib and cisplatin in vitro (Figure 1H).

Strikingly, the expression of KAT6B, paralogous to KAT6A, was not much higher in DTP cells than in apoptotic cells in response to PARPi treatment (Figure 1G). Furthermore, we found that KAT6A knockout, but not catalytic inhibition of KAT6A using a small‐molecule inhibitor (WM1119),^[^ ^6^, ^7^ ^]^ restored the PARPi sensitivity of ovarian cancer cells (Figure 1I–K; Figure S1D, Supporting Information). Moreover, in a xenograft ovarian tumor model treated with olaparib and cisplatin, KAT6A knockout, but not WM1119 treatment, impaired PARPi resistance in ovarian cancer. The KAT6A knockout group exhibited slower tumor growth and longer survival after PARPi treatment than the other groups (Figure 1L,M). Additionally, WM1119 treatment, both in vivo and in vitro, did not influence PARPi sensitivity (Figure 1I–M). In KAT6A‐KO SKOV3‐R or UWB1.289‐R cells, re‐expression of KAT6A^C543G/G657E^ (KAT‐deficient mutants) also rendered the cells resistant to PARPi^[^ ^10^ ^]^ (Figure 1I–K). KAT6A^C543G/G657E^ exhibited the same PARPi resistance‐promoting effects in vivo as KAT6A‐WT (Figure 1L,M; Figure S1J, Supporting Information). Moreover, knockout of KAT6A, but not of KAT6B, restored sensitivity to PARPi treatment, indicating that KAT6A may promote PARPi resistance independent of its catalytic function (Figure S1D–I, Supporting Information). As an acetyltransferase, KAT6A promotes the development of several cancers by acetylating histone H3 (H3K23ac).^[^ ^7^, ^10^, ^11^ ^]^ However, we observed no significant differences in the acetylation levels of KAT6A substrates between PARPi‐resistant and PARPi‐sensitive ovarian cancer cells (Figure S1K, Supporting Information).

Collectively, these findings indicate that KAT6A promotes PARPi resistance in ovarian cancer cells independent of its catalytic function.

### 2.2 KAT6A LLPS Enhances the Interaction of KAT6A and PARP1 in PARPi‐Resistant Ovarian Cancer Cells

**Figure 2:**
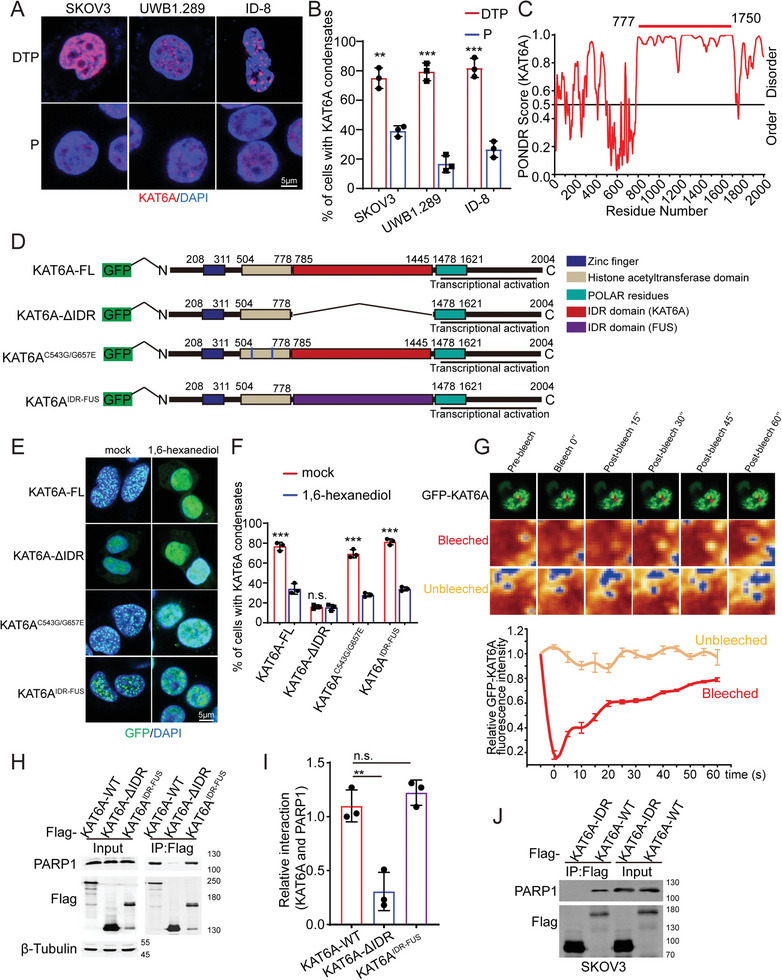
Liquid‐liquid phase separation of KAT6A enhances interaction of KAT6A and PARP1 in PARPi‐resistant ovarian cancer cells. A,B) Biomolecular condensates of KAT6A in PARPi‐resistant ovarian cancer cells (DTP cells) and parental ovarian cancer cells (P cells). ≥10 condensates were identified as cells with KAT6A condensates in this study. The representative images are shown in A, and quantification is shown in B. The number of cells with KAT6A condensates per 100 cells from 3 independent experiments was recorded and analyzed (Unpaired t‐test). C) The disorder and order sequences of KAT6A analyzed using PONDR database. D) The pattern diagram of GFP tagged full‐length KAT6A (KAT6A ‐FL), IDR domain deleted KAT6A protein (KAT6A‐ΔIDR), KAT‐deficient mutated KAT6A (KAT6A^C543G/G657E^) and chimeric KAT6A protein (KAT6A^IDR‐FUS^). E,F) The biomolecular condensates (diameters greater than 0.5 µm were defined as KAT6A puncta) of KAT6A ‐FL, KAT6A^C543G/G657E^, and KAT6A^IDR‐FUS^ were destroyed by 1,6‐hexanediol treatment. Loss of IDR domain impairs formation of condensates. The representative images are shown in E, and quantification is shown in F. The number of cells with KAT6A condensates per 100 cells from 3 independent experiments was recorded and analyzed (Unpaired t‐test). See also Videos S1 and S2 (Supporting Information). G) The FRAP assays demonstrated the fluidity of KAT6A condensates. Upper, representative images of KAT6A condensates in FRAP assays. Lower, statistical analysis of GFP‐KAT6A fluorescence intensity in FRAP assays. H,I) Deletion of IDR domain impairs the interaction of KAT6A and PARP1 which can be rescued by insertion of IDR domain from FUS. Quantification of H is shown in I (one‐way ANOVA followed by Dunnett‐t multiple‐comparison test, *n* = 3 per group). J) IDR domain of KAT6A doesn't interact with PARP1. All data are expressed as mean ± SD. **P < 0.01; ***P < 0.001; n.s. denotes no signification.

Immunofluorescence assays revealed that KAT6A formed more biomolecular condensates in PARPi‐resistant ovarian cancer than in parental cells (**Figure** **2**A,B; Video S1, Supporting Information). This observation led us to hypothesize that condensates of KAT6A promote PARPi resistance in ovarian cancer. We analyzed the KAT6A protein sequence using the POUND database and identified a region with a high disorder score within its amino acid sequence (aa 778–1478)^[^ ^12^ ^]^ (Figure 2C), indicating the potential characteristics of LLPS. To detect the function of KAT6A LLPS, truncated KAT6A with impaired LLPS was used in subsequent studies. Because the C‐terminus of KAT6A is essential for its transcriptional activation, and POLAR residues are important for protein structure or stability,^[^ ^13^ ^]^ a smaller range of amino acids (aa 778–1478) was deleted to preserve its transcriptional activation and POLAR residues (Figure 2C,D). In this study, we identified a version of KAT6A lacking amino acid residues 778–1478 as an internal disordered region (IDR)‐deleted KAT6A (KAT6A‐ΔIDR). Deletion of amino acids 778–1478 in KAT6A did not significantly influence the formation of the BRPF1‐KAT6A/KAT6B complex, acetyltransferase activity, transcriptional activation of KAT6A, or its ability to bind various proteins, as previously reported^[^ ^7^, ^13^, ^14^ ^]^ (Figure S2A–E, Supporting Information). KAT6A‐ΔIDR also induced resistance to cisplatin treatment by acetylating COP1 and stabilizing β‐catenin, similar to KAT6A‐WT in ovarian cancer cells^[^ ^6^ ^]^ (Figure S2D,E, Supporting Information). Moreover, mass spectrometry analysis following co‐immunoprecipitation showed that most KAT6A‐associated proteins can also bind KAT6A‐ΔIDR (Table S1, Supporting Information). Proteins that bound to KAT6A‐WT but not to KAT6A‐ΔIDR are irrelevant to PARPi resistance, DNA damage repair, and oncogenesis (Table S2 Figure S2F, Supporting Information). Consequently, KAT6A‐ΔIDR is a functional protein with impaired LLPS.

We next overexpressed GFP‐labeled KAT6A and KAT6A‐ΔIDR in SKOV3‐R cells (Figure 2D; Figure S2G, Supporting Information). The biomolecular condensates of KAT6A were destroyed by 1,6‐hexanediol, a compound commonly used to reverse LLPS,^[^ ^15^ ^]^ in SKOV3‐R cells (Figure 2E,F). This finding demonstrates that the KAT6A droplets are reversible phase‐separated condensates. In addition, KAT6A^C543G/G657E^ also exhibited the LLPS characteristics seen in KAT6A‐WT (Figure 2E,F; Figure S2G, Supporting Information), and its function as a histone acetyltransferase did not influence the formation of biomolecular condensates. However, KAT6A‐ΔIDR did not form biomolecular condensates in SKOV3‐R cells (Figure 2E,F; Figure S2G and Video S2, Supporting Information). To confirm our finding, we employed the fluorescence recovery rate after photobleaching (FRAP) method.^[^ ^16^ ^]^ We found that GFP‐KAT6A foci recovered fluorescence 60 s after photobleaching (Figure 2G). Next, we replaced the IDR domain of KAT6A with the IDR domain of FUS, which is known to form liquid‐like droplets as a disordered protein that does not interact with PARP1^[^ ^17^ ^]^ (Figure S2C,D, Supporting Information) and obtained a new chimeric KAT6A protein (KAT6A^IDR‐FUS^) (Figure 2D; Figure S2G, Supporting Information). KAT6A^IDR‐FUS^ also formed biomolecular condensates that were destroyed by 1,6‐hexanediol in SKOR3‐R cells (Figure 2E,F). These data demonstrate that the LLPS of KAT6A is enhanced in PARPi‐resistant ovarian cancer cells and that this process was dependent on the IDR sequence.

The interaction between KAT6A and PARP1 was increased in PARPi‐resistant ovarian cancer cells, as observed above; therefore, we further analyzed the relationship between KAT6A LLPS and the binding of the two proteins. The disruption of LLPS impaired the interaction between KAT6A and PARP1 (Figure 2H,I). To eliminate the effects of LLPS on the interaction between KAT6A and PARP1, we restored the LLPS of KAT6A using the IDR domain of FUS. KAT6A^IDR‐FUS^ also bound more PARP1 than KAT6A‐ΔIDR (Figure 2H,I), indicating that KAT6A LLPS enhances the interaction of KAT6A and PARP1. Additionally, PARP1 did not bind to the IDR domain of KAT6A (KAT6A‐IDR) (Figure 2J). Thus, the impairment of the interaction between KAT6A‐ΔIDR and PARP1 is due to LLPS disruption rather than a decreased availability of binding sites. Consequently, the increased interaction between KAT6A and PARP1 primarily resulted from the LLPS of KAT6A.

Taken together, KAT6A LLPS was enhanced in PARPi‐resistant ovarian cancer cells, and strengthens the interaction between KAT6A and PARP1.

### 2.3 KAT6A LLPS Impairs PARPi‐Induced PARP1 Trapping

**Figure 3:**
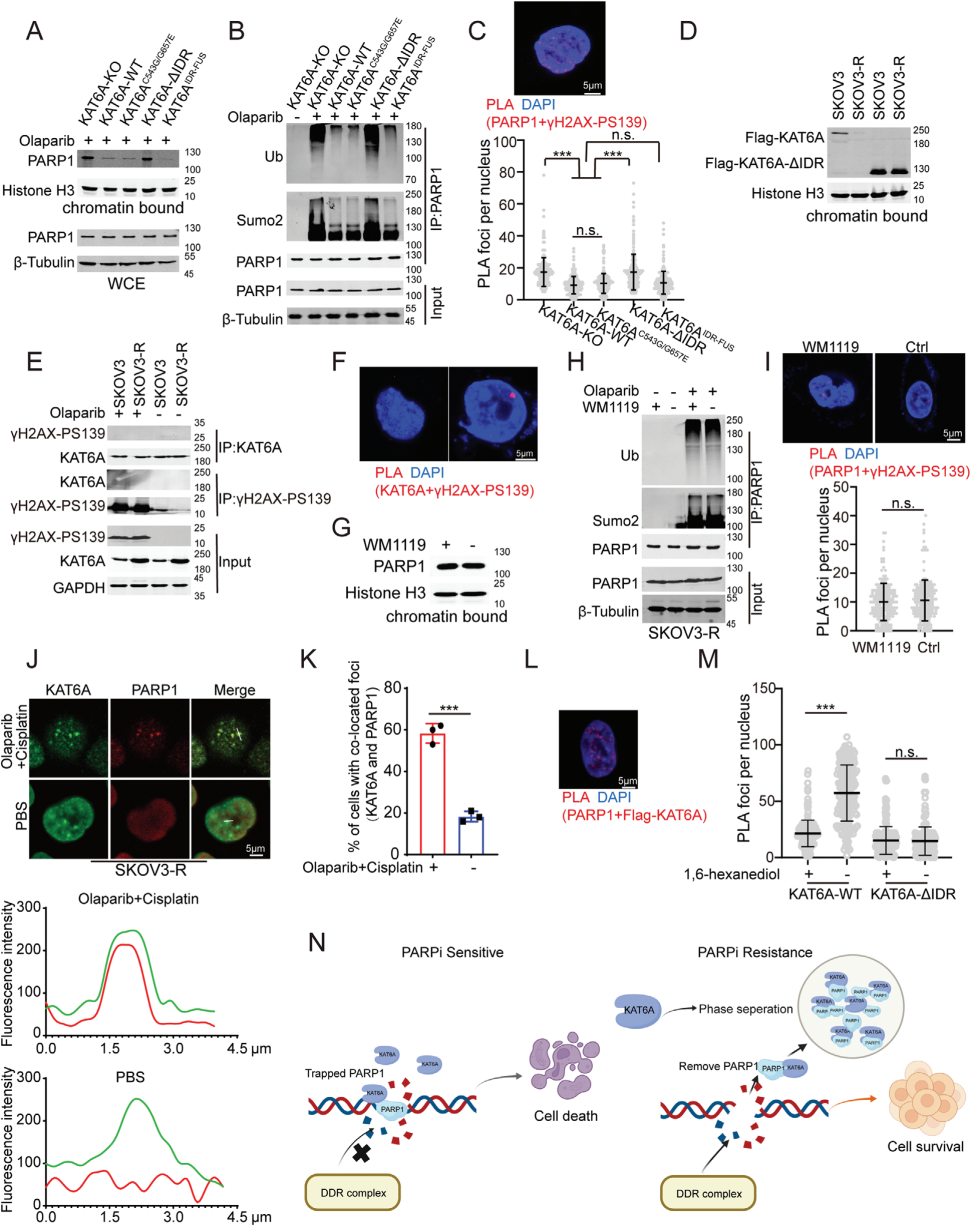
LLPS of KAT6A impairs PARP1 trapping induced by PARPi. A) PARP1 trapping in SKOV3‐R cells of indicated genotypes treated with olaparib. KAT6A‐WT, KAT6A^C543G/G657E^ or KAT6A^IDR‐FUS^ but not KAT6A‐ΔIDR overexpression impairs PARP1 trapping. Total PARP1 proteins were also detected using whole cell extract (WCE). Chromatin‐bound proteins were extracted and analyzed using the indicated antibodies. B) KAT6A‐ΔIDR but not KAT6A‐WT, KAT6A^C543G/G657E^ or KAT6A^IDR‐FUS^ decreases the level of sumoylation and ubiquitination modification of PARP1 induced by olaparib in SKOV3‐R. C) Representative image of PLA for endogenous PARP1 and γH2AX‐PS139 in SKOV3‐R cells is shown in upper, and the quantification is shown in lower. PARP1–γH2AX‐PS139 PLA (anti‐PARP1 and anti‐γH2AX‐PS139) in SKOV3‐R cells expressing KAT6A‐WT, KAT6A^C543G/G657E^, KAT6A‐ΔIDR, or KAT6A^IDR‐FUS^ during treatment of olaparib. KAT6A‐WT, KAT6A^C543G/G657E^, and KAT6A^IDR‐FUS^ overexpression decreased significantly PARP1–γH2AX‐PS139 PLA, but KAT6A‐ΔIDR didn't. the data were analyzed using one‐way ANOVA followed by Tukey's multiple‐comparison test, *n* = 200 per group. Representative results from one of three independent experiments are shown. D) The decreased WT‐KAT6A is bound on the chromatin in SKOV3‐R compared to SKOV3, but no significant difference in KAT6A‐ΔIDR bound on the chromatin between SKOV3‐R and SKOV3 cells. E) Co‐IP assays showed that of KAT6A doesn't interact with γH2AX‐PS139 with or without olaparib treatment. F) Representative image of a PLA for endogenous KAT6A and γH2AX‐PS139 in SKOV3‐R cells. G) PARP1 trapping in SKOV3‐R cells with or without WM1119 treatment in SKOV3‐R cells with laparib and cisplatin treatment. H) WM1119 has no influence on the level of sumoylation and ubiquitination modification of PARP1 induced by olaparib in SKOV3‐R. I) The effects of WM1119 on interaction of PARP1 and γH2AX‐PS139 in SKOV3‐R cells with olaparib and cisplatin treatment. The representative image of PLA for endogenous PARP1 and γH2AX‐PS139 is shown in upper, and quantification is shown in lower (Unpaired t‐test, *n* = 200 per group). J) More PARP1 proteins are co‐located with KAT6A droplets during olaparib and cisplatin treatment. The representative images of PARP1 and KAT6A by IF assays are shown in upper, and the quantification of fluorescence intensity from the indicated regions (white line) in upper is shown in lower. K) Cell with ≥5 co‐located foci of KAT6A and PARP1 proteins from J was identified as cells with co‐located foci. The number of cells with co‐located foci per 100 cells from 3 independent experiments was recorded and analyzed (Unpaired t‐test). L,M) The effects of 1,6‐hexanediol on interaction of PARP1 and KAT6A or KAT6A‐ΔIDR in SKOV3‐R cells with olaparib and cisplatin treatment. The representative image of PLA is shown in L, and quantification is shown in M (Unpaired t‐test, *n* = 200 per group). N) Schematic representation that KAT6A LLPS impairs PARP1 trapping. The image was created by biorender.com. All data are expressed as mean ± SD. ***P < 0.001; n.s. denotes no signification.

Droplets formed by LLPS can store high concentrations of proteins and release them into the cell environment when required to regulate the concentrations of related proteins in the cell.^[^ ^18^ ^]^ In addition, LLPS mediates the localization of certain proteins to existing phase‐separation droplets or membrane‐free organelles. Both KAT6A LLPS and the interaction between KAT6A and PARP1 were enhanced in the PARPi‐resistant ovarian cancer cells (Figures 1 and 2). Thus, we speculated that the LLPS of KAT6A may mediate the localization of PARP1 by adsorbing PARP1 into liquid‐like droplets. As shown in **Figure** **3**A, KAT6A, but not KAT6A‐ΔIDR, reduced the amount of PARP1 bound to chromatin, which contributes to the cytotoxic effect of PARPi (PARP1 trapping). Sumoylation and ubiquitination levels of PARP1 are reportedly enhanced when the PARP1 protein is trapped; as such, this can be considered a biomarker for trapped PARP1.^[^ ^19^ ^]^ Re‐expression of KAT6A, but not KAT6A‐ΔIDR, reduces sumoylation and ubiquitination levels of PARP1 (Figure 3B). Moreover, proximity ligation assays (PLA) were performed, showing that re‐expression of KAT6A impaired the colocalization of PARP1 and DNA break sites (ascertained by γH2AX‐PS139 positivity; Figure 3C). Furthermore, we found that inhibiting KAT6A LLPS by deleting IDR could restore PARP1 trapping, but KAT6A^IDR‐FUS^ or KAT6A^C543G/G657E^ could not (Figure 3A–C). These data suggest that the condensation of KAT6A attenuates PARP1 trapping during PARPi treatment.

KAT6A, known as a histone acetyltransferase, binds to and modifies chromatin in cancer cells.^[^ ^11^, ^20^ ^]^ In this study, we observed lower levels of chromatin‐bound KAT6A‐WT in PARPi‐resistant ovarian cancer cells compared to PARPi‐sensitive ones (Figure 3D). Additionally, KAT6A rarely co‐localized with DNA break sites in SKOV3‐R cells compared to SKOV3 cells (Figure 3E,F). Conversely, KAT6A‐ΔIDR did not exhibit a significant difference in its ability to bind chromatin in either PARPi‐resistant or PARPi‐sensitive ovarian cancer cells (Figure 3D). Notably, treatment with WM1119 had no impact on the separation of PARP1 from DNA break sites or on the sumoylation and ubiquitination modifications of PARP1 (Figure 3G–I). Furthermore, KAT6A^C543G/G657E^ impaired PARP1 trapping (Figure 3A–C), suggesting that KAT6A attenuates PARP1 trapping independent of its catalytic function.

Given that KAT6A LLPS reduces its capacity to bind chromatin, and deletion of the IDR domain from KAT6A disrupts the interaction between KAT6A and PARP1 in PARPi‐resistant ovarian cancer cells, our next inquiry focused on whether KAT6A impedes PARP1 trapping via LLPS. We observed increased co‐localization of PARP1 proteins with KAT6A droplets upon PARPi treatment (Figure 3J,K). Furthermore, treatment with 1,6‐hexanediol hindered the interaction between KAT6A and PARP1 but had no effect on the interaction between KAT6A‐ΔIDR and PARP1 (Figure 3L,M), suggesting that PARP1 is induced to enter KAT6A droplets during PARPi treatment.

Collectively, these findings suggest that KAT6A sequesters PARP1 away from DNA break sites, impairing PARP1 trapping during PARPi treatment of PARPi‐resistant ovarian cancer cells (Figure 3N).

### 2.4 Inhibiting LLPS, but not the Catalytic Activity of KAT6A, Represses PARPi Resistance in Ovarian Cancer Cells

**Figure 4:**
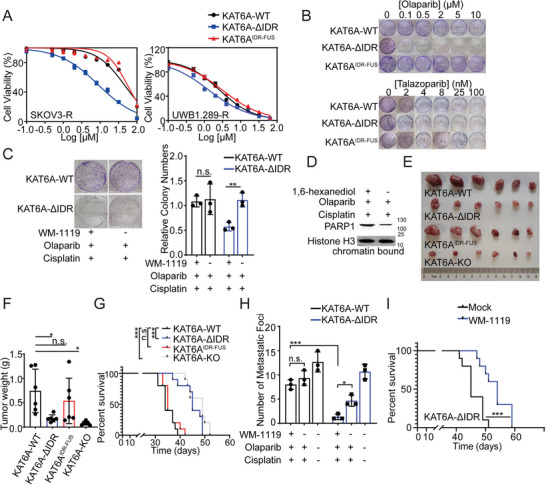
Inhibiting LLPS but not catalytic activity of KAT6A represses PARPi resistance in ovarian cancer cells. A) UWB1.289‐R and SKOV3‐R cells of indicated genotypes were treated with olaparib at increasing concentrations for 72 h, after which cell viability was measured using Cell Titer‐Glo luminescent assay (replicates, *n* = 3; symbols represent averages of experimental replicates; error bars indicate SEM). Representative results from one of three independent experiments are shown. B) Clonogenic assay illustrating the restoration of PARPi sensitivity (olaparib or Talazoparib) in the KAT6A‐ΔIDR SKOV3‐R cells but not KAT6A^IDR‐FUS^ SKOV3‐R cells. Representative results from one of three independent experiments are shown. C) WM‐1119 restores PARPi sensitivity in KAT6A‐ΔIDR but not KAT6A‐WT SKOV3‐R cells. The representative images are shown in left and statistical analysis is shown in right (Unpaired *t*‐test, *n* = 3 per group). D) PARP1 trapping was impaired by 1, 6‐hexanediol. E) SKOV3‐R cells of indicated genotypes were injected subcutaneously into the hind flanks of nu/nu mice. Mice were treated with olaparib and cisplatin during week 2–4. The representative image of tumor 4 weeks post injection is shown. F) Quantification of E (one‐way ANOVA followed by Tukey's multiple‐comparison test, *n* = 6 per group). G) Survival of C57BL/6 mice injected i.p. with ID8‐R cells of indicated genotypes (Log‐rank test, *n* = 10 per group). Mice were treated with olaparib and cisplatin during week 2–4. Deleting IDR of KAT6A or KAT6A‐KO restores sensitivity of ovarian cancer to PARPi in mice. H) Balb/c nude mice were injected i.p. with SKOV3‐R cells of indicated genotypes and mice were treated using olaparib and cisplatin during week 2–4. And the metastatic foci were counted (one‐way ANOVA followed by Tukey's multiple‐comparison test, n = 3 per group). WM‐1119 treatment reduced the number of metastatic foci of KAT6A‐ΔIDR group but had no influence on metastatic foci of KAT6A‐WT group. I) Survival of Balb/c nude mice injected i.p. with KAT6A‐ΔIDR SKOV3‐R cells (Log‐rank test, *n* = 10 per group). Mice were treated with olaparib and cisplatin with or without WM‐1119 (60 mg kg^−1^) during week 2–4. Data of C, F, and H are expressed as mean ± SD. *P < 0.05; **P < 0.01; ***P < 0.001; n.s. denotes no signification.

Most clinical PARPis not only inhibit catalytic activity by binding to the NAD^+^‐binding site^[^ ^21^ ^]^ but also induce PARP1 trapping.^[^ ^22^ ^]^ Recently, the latter characteristic was shown to be a key driver of PARPi‐mediated cytotoxicity.^[^ ^4^, ^22^ ^]^ Here, we found that KAT6A LLPS enhances the interaction between KAT6A and PARP1 proteins and impairs PARP1 trapping. Therefore, we examined the influence of KAT6A LLPS on PARPi resistance in ovarian cancer. KAT6A‐ΔIDR, whose LLPS is impaired, significantly improved sensitivity to PARPi in ovarian cancer cells (**Figure** **4**A,B). However, both KAT6A^IDR‐FUS^, whose LLPS was rescued by IDR from FUS, and KAT6A^C543G/G657E^ enhanced PARPi resistance in ovarian cancer cells (Figures 1H–J and 4A,B), indicating that KAT6A LLPS, but not its catalytic function, counteracted the effects of PARPi. Furthermore, WM‐1119 treatment restored PARPi sensitivity in KAT6A‐ΔIDR but not KAT6A‐WT SKOV3‐R cells (Figure 4C). Catalytic inhibition of KAT6A impaired PARPi resistance in KAT6A‐ΔIDR SKOV3‐R cells, but the effect was neutralized in KAT6A‐WT SKOV3‐R cells. Owing to the key role of PARP1 trapping in PARPi‐mediated cytotoxicity, the catalytic function of KAT6A can be ignored compared with that of KAT6A LLPS in PARPi resistance. We further disrupted the LLPS of KAT6A using 1,6‐hexanediol, restoring PARP1 trapping (Figure 4D). These data demonstrate that KAT6A LLPS promotes PARPi resistance independent of its catalytic function and that inhibition of KAT6A LLPS is a potential therapeutic strategy for PARPi‐resistant ovarian cancer.

To validate our findings, we constructed in vivo xenograft tumor models and assessed the influence of KAT6A condensates on PARPi resistance. As shown in Figure 4E–G, KAT6A‐ΔIDR decreased the growth of tumors and prolonged the survival of mice undergoing PARPi treatment. However, KAT6A^IDR‐FUS^ and KAT6A^C543G/G657E^ promoted PARPi resistance in vivo and shortened the lifespans of mice.

Ascites are an important complication of ovarian cancer, indicating the metastasis and development of tumors.^[^ ^23^ ^]^ Next, we constructed a metastatic model in C57BL/6 mice using ID‐8‐R cells of the indicated genotypes.^[^ ^24^ ^]^ It was found that KAT6A‐KO or KAT6A‐ΔIDR decreased the number of metastatic foci and repressed the formation of ascites under olaparib and cisplatin treatment (Figure 4H; Figure S3A, Supporting Information). In addition, WM‐1119 treatment enhanced the cytotoxic effects of olaparib and cisplatin and prolonged the survival of mice in the KAT6A‐ΔIDR group, but not in the KAT6A‐WT group (Figure 4I; Figure S3B, Supporting Information). WM‐1119 decreased the number of metastatic foci and amount of ascites fluid formation in the KAT6A‐ΔIDR group, but WM‐1119 had no effects on the development of KAT6A‐WT ovarian cancer (Figure 4I; Figure S3B, Supporting Information), demonstrating that KAT6A promotes PARPi resistance independent of its catalytic function.

Overall, this evidence indicates that targeting the LLPS of KAT6A represses the development of PARPi‐resistant ovarian cancer cells.

### 2.5 Impaired PARP1 Trapping Induced by LLPS of KAT6A Enhances DNA Damage Repair (DDR) during PARPi Treatment

**Figure 5:**
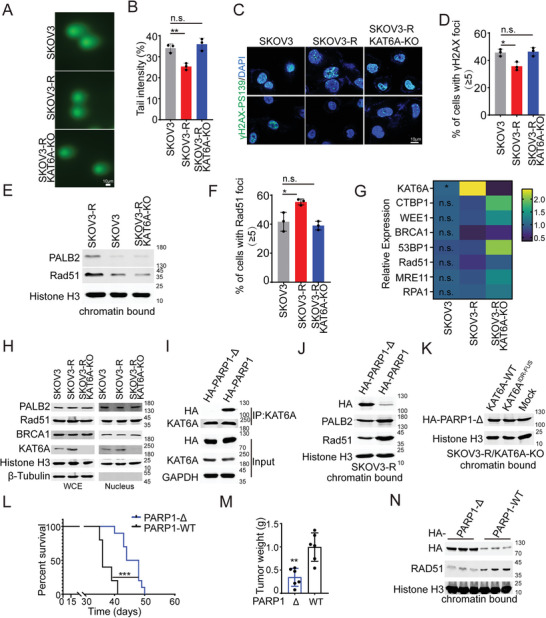
Impaired PARP1 trapping induced by LLPS of KAT6A enhances DDR during PARPi treatment. A) Representative images of comet tails in SKOV3, SKOV3‐R, and KAT6A‐KO SKOV3‐R cells 48 h post treatment with cisplatin. Scale bars, 10 µm. B) Quantification of comet tail intensity in A (one‐way ANOVA followed by Tukey's multiple‐comparison test). The experiment was performed and analyzed in triplicate. C) Representative images of γH2AX‐PS139 foci in SKOV3, SKOV3‐R, and KAT6A‐KO SKOV3‐R cells 48 hours post treatment with cisplatin. Scale bars, 10 µm. D) Quantification of γH2AX‐PS139 foci in C (one‐way ANOVA followed by Tukey's multiple‐comparison test). The experiment was performed and analyzed in triplicate. E) the amount of RAD51 and PALB2 proteins in chromatin fractionation from SKOV3, SKOV3‐R, and KAT6A‐KO SKOV3‐R cells 48 h post treatment with cisplatin. F) Quantification of RAD51 foci in SKOV3, SKOV3‐R, and KAT6A‐KO SKOV3‐R cells 48 h post treatment with cisplatin (one‐way ANOVA followed by Tukey's multiple‐comparison test). The experiment was performed and analyzed in triplicate. G,H) qRT‐PCR assays (G, one‐way ANOVA, *n* = 3 per group) and Immunoblotting assays (H) show no significant difference in key factors of DDR between indicated groups. I) Deletion of N‐terminal 10 amino acids (MAESSDKLYR) in PARP1 (PARP1‐Δ) impairs the interaction of KAT6A and PARP1. J) More PARP1‐Δ was trapped by olaparib and impaired DDR in PARP1‐Δ SKOV3‐R cells. K) Overexpression of KAT6A or KAT6A^IDR‐FUS^ has no influence on PARP1‐Δ trapping. L) survival of mice injected i.p. with SKOV3‐R cells of PARP1‐Δ or PARP1‐WT. Mice were treated using olaparib and cisplatin during week 2–4. PARP1‐Δ inhibits the development of ovarian cancer compared to PARP1‐WT (Log‐rank test, n = 10 per group). M) SKOV3‐R cells of PARP1‐Δ or PARP1‐WT were injected subcutaneously into the hind flanks of nu/nu mice. Mice were treated using olaparib and cisplatin during week 2–4. Quantification of the tumor weight at week 4 is shown in M (Unpaired t‐test, *n* = 6 per group). N) Enhanced PARP1‐Δ trapping and repressed DDR were detected in PARP1‐Δ tumors than that in PARP1‐WT tumors in vivo. All data are expressed as mean ± SD. *P < 0.05; **P < 0.01; ***P < 0.001; n.s. denotes no signification.

Enhanced DDR is an essential signature of PARPi‐resistant cancer cells.^[^ ^1^ ^]^ In addition, PARP1 trapping could create DNA break sites occupied by PARP1 proteins and inhibit the subsequent DDR by preventing functional molecules (for example, RAD51 and PALB2) from binding to the DNA lesions.^[^ ^4^, ^25^ ^]^ Here, DDR ability was determined by comet assays and quantification of γH2AX‐PS139 foci. SKOV3‐R cells showed improved DDR capacity compared to primary SKOV3 cells, which were blocked by KAT6A KO (**Figure** **5**A–D; Figure S3C, Supporting Information). We also found that more RAD51 and PALB2 proteins bound to DNA break sites in SKOV3‐R than in SKOV3 cells, which was also observed in KAT6A KO SKOV3‐R cells (Figure 5E,F). Furthermore, KAT6A is a transcription coactivator that acetylates histones.^[^ ^7c^ ^]^ To determine the mechanism by which KAT6A knockout impairs DDR in PARPi‐resistant ovarian cancer cells, we quantified the expression of key factors involved in DDR. We found no significant differences between the indicated groups at the mRNA or protein levels (Figure 5G,H). RNA‐seq was also performed to exclude the involvement of other molecular mechanisms that enhance DDR capacity. No pathways directly related to DDR were significantly enhanced in SKOV3‐R cells (Figure S4A,B, Supporting Information).

Next, we assessed whether the LLPS of KAT6A enhanced DDR by repressing PARP1 trapping. Re‐expression of KAT6A or KAT6A^IDR‐FUS^ rescued the DDR of SKOV3‐R cells, but KAT6A‐ΔIDR showed no significant effect on DDR during PARPi treatment (Figure S3D, Supporting Information). Furthermore, we found that the 10 N‐terminal amino acids (MAESSDKLYR) of PARP1 were indispensable for the interaction between KAT6A and PARP1 by truncating the PARP1 protein (Figure 5I; Figure S5A,D, Supporting Information). To delineate the relationship between the LLPS of KAT6A and the enhancement of DDR, we deleted these N‐terminal amino acids (PARP1‐Δ), which are important for binding KAT6A (Figure 5I). Compared to PARP1‐WT, PARP1‐Δ significantly increased the content of PARP1 trapped on the DNA lesions and subsequently decreased the content of functional molecules of DDR bound to DNA break sites during cisplatin + olaparib treatment (Figure 5J). Moreover, overexpression of KAT6A or KAT6A^IDR‐FUS^ had no effect on PARP1‐Δ trapping in SKOV3‐R cells (Figure 5K).

Finally, we assessed the influence of the disruption of KAT6A and PARP1 interaction on tumor cell survival in vivo. The PARP1‐Δ group showed higher sensitivity to PARPi treatment and longer survival than the PARP1‐WT group (Figure 5L,M; Figure S5E, Supporting Information). Furthermore, PARP‐Δ possesses a greater ability to trap PARP1 within DNA lesions and inhibit the subsequent DDR (Figure 5N).

Collectively, these results indicate that the LLPS of KAT6A enhances DDR by impairing PARPi‐mediated PARP1 trapping, and that disruption of the interaction between KAT6A and PARP1 could neutralize this effect.

### 2.6 APEX1 is Essential for KAT6A LLPS and the Interaction of KAT6A and PARP1

**Figure 6:**
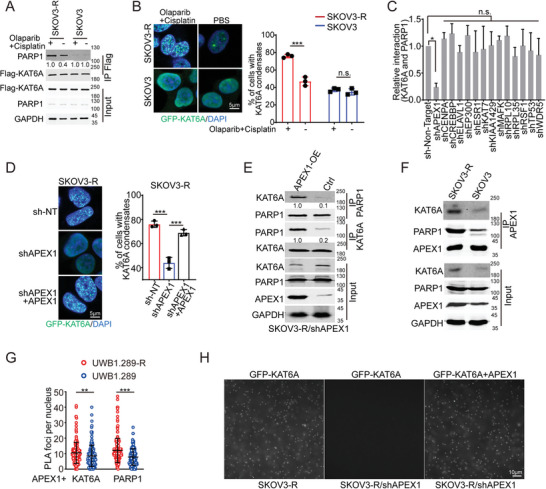
APEX1 is essential for LLPS of KAT6A. A) KAT6A was overexpressed to the same level in SKOV3 or SKOV3‐R cells. Co‐IP assay shows that more interaction of KAT6A and PARP1 in SKOV3‐R but not SKOV3 cells responding to olaparib and cisplatin treatment. The experiment was performed and analyzed in triplicate. B) KAT6A was overexpressed to the same level in SKOV3 or SKOV3‐R cells. More KAT6A condensates were formed in SKOV3‐R but not SKOV3 cells responding to olaparib and cisplatin treatment. The representative images are shown in left and statistical analysis is shown in right (Unpaired t‐test, *n* = 3 per group). C) Repression of APEX1 but not the other indicated gene impairs the interaction of KAT6A and PARP1 in PARPi‐resistant ovarian cancer cells. D) APEX1 KD impaired the formation of KAT6A condensates, which can be rescued by APEX1 overexpression. The representative images are shown in left and statistical analysis is shown in right (Unpaired *t*‐test, *n* = 3 per group). E) Re‐expression of APEX1 enhanced the interaction of KAT6A and PARP1 in PARPi‐resistant ovarian cancer cells. The experiment was performed and analyzed in triplicate. F,G) Co‐IP assays (F) and PLA assays (G, Unpaired *t*‐test, *n* = 200 per group) showed that the KAT6A‐PARP1‐APEX1complex is more stable in SKOV3‐R cells than that in SKOV3 cells. H) KAT6A condensation in vitro. EGFP tagged KAT6A proteins from SKOV3‐R cells with or without APEX1 knockdown were purified and quantified. Representative fluorescence images of 1.5 µm EGFP‐KAT6A from indicated cells were obtained immediately after mixing with or without 10% PEG. APEX1 knockdown impairs the formation of KAT6A condensates that were rescued by adding recombinant APEX1 proteins. Scale bars, 10 µm. Representative results from one of three independent experiments are shown. All data are expressed as mean ± SD. **P < 0.01; ***P < 0.001; n.s. denotes no signification.

To explore the molecular mechanisms of enhanced KAT6A LLPS and the interaction between KAT6A and PARP1 in PARPi‐resistant ovarian cancer cells, poly(ADP‐ribose) (PAR) chains on KAT6A and acetylated PARP1 were detected. No significant differences were found between PARPi‐resistant and primary ovarian cancer cells (Figure S6A,B, Supporting Information). To validate whether KAT6A LLPS was induced by a gap in the KAT6A expression level (Figure 2), the same amount of KAT6A was overexpressed in primary or PARPi‐resistant ovarian cancer cells, and KAT6A LLPS and the interaction of KAT6A with PARP1 were tested (**Figure** **6**A). Both KAT6A droplets and the interaction of KAT6A with PARP1 increased in response to olaparib + cisplatin treatment in PARPi‐resistant cancer cells but not in primary ovarian ones (Figure 6A,B), indicating that supplemental molecular mechanisms promote the formation of KAT6A LLPS and the interaction of KAT6A with PARP1 in PARPi‐resistant ovarian cancer cells.

Next, proteins that interacted with both KAT6A and PARP1 were searched and screened using a public database (BioGRID) and our previous data.^[^ ^6^ ^]^ Fourteen proteins that bind to both KAT6A and PARP1 were identified (Figure S6C, Supporting Information). We knocked down the indicated proteins in SKOV3‐R cells using siRNA (Figure S6D, Supporting Information), and detected the interaction between KAT6A and PARP1 during Olaparib + cisplatin treatment. The interaction between KAT6A and PARP1 was impaired in APEX1‐KD SKOV3‐R cells (Figure 6C; Figure S6E,F, Supporting Information). In addition, deletion of APEX1 impaired the formation of KAT6A condensates, which could be rescued by APEX1 overexpression in SKOV3‐R cells (Figure 6D). Re‐expression of APEX1 rescued the interaction between KAT6A and PARP1 (Figure 6E), indicating that APEX1 plays an important role in the interaction between KAT6A and PARP1. Moreover, the expression of APEX1 in PARPi‐resistant ovarian cancer cells was much higher than that in primary ovarian cancer cells (Figure 6F). Moreover, the interaction between APEX1 and KAT6A or PARP1 was enhanced in PARPi‐resistant ovarian cancer cells during olaparib + cisplatin treatment (Figure 6F,G), indicating the formation of a more stable complex containing KAT6A, PARP1, and APEX1.

To further confirm the KAT6A LLPS‐promoting effects of APEX1, we purified EGFP‐tagged KAT6A proteins from SKOV3‐R cells with or without APEX1 knockdown, and detected KAT6A condensation in vitro.^[^ ^26^ ^]^ APEX1 KD impaired the formation of KAT6A condensation in vitro, and KAT6A condensation was rescued by supplementation with APEX1 proteins (Figure 6H), demonstrating the essential role of APEX1 in KAT6A LLPS.

Overall, these data indicate that APEX1 is essential for the LLPS of KAT6A and the interaction of KAT6A with PARP1 in PARPi‐resistant ovarian cancer cells.

### 2.7 APEX1 Promotes PARPi Resistance by Enhancing KAT6A LLPS and the Interaction of KAT6A and PARP1

**Figure 7:**
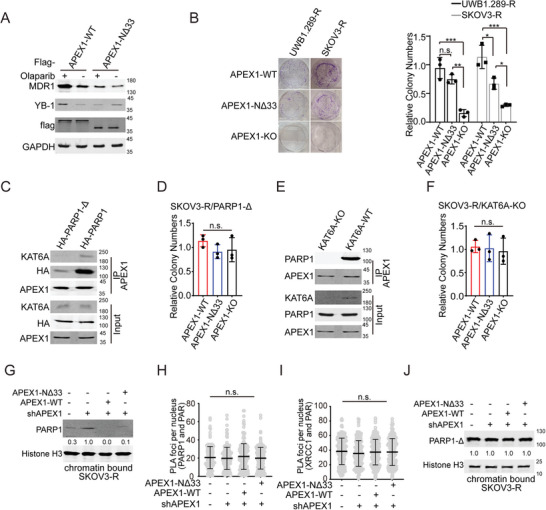
APEX1 promotes PARPi resistance by enhancing KAT6A LLPS and the interaction of KAT6A and PARP1. A) APEX1‐WT rather than APEX1‐NΔ33 expression of genes related to multidrug resistance responding to PARPi treatment in SKOV3‐R cells. B) APEX1‐WT or APEX1‐NΔ33 overexpression can promote PARPi resistance. The representative images are shown on left and statistical analysis is shown in right (one‐way ANOVA followed by Tukey's multiple‐comparison test, *n* = 3 per group). Representative results from one of three independent experiments are shown. C) The instability of KAT6A‐PARP1‐APEX1complex in PARP1‐Δ SKOV3‐R cells. D) Re‐expression of APEX1‐WT or APEX1‐NΔ33 has no effects on PARPi resistance of PARP1‐Δ SKOV3‐R cells (one‐way ANOVA, *n* = 3 per group). E) The instability of KAT6A‐PARP1‐APEX1complex in KAT6A‐KO SKOV3‐R cells. F) Re‐expression of APEX1‐WT or APEX1‐NΔ33 has no effects on PARPi resistance in KAT6A‐KO SKOV3‐R cells (one‐way ANOVA, *n* = 3 per group). G) APEX1 knockdown decreases the amount of PARP1 trapped on chromatin, and both APEX1‐WT and APEX1‐NΔ33 rescue PARP1 trapping induced by PARPi treatment. Representative results from one of three independent experiments are shown. H,I) The detection of PAR chains on PARP1(H) or XRCC1 (I) using PLA assays. PLA assays were performed by anti‐PAR and anti‐ PARP1(H) or anti‐XRCC1 (I), and the PLA foci were quantified and analyzed (one‐way ANOVA, *n* = 200 per group). J) Regulation of APEX1 has no influence on the amount of PARP1 trapped on chromatin. Representative results from one of three independent experiments are shown. All data are expressed as mean ± SD. *P < 0.05; **P < 0.01; ***P < 0.001; n.s. denotes no signification.

APEX1 is a member of the apurinic/apyrimidinic (AP) endodeoxyribonuclease family and exhibits critical functions in the DNA base excision repair (BER) pathway.^[^ ^27^ ^]^ APEX1 overexpression is often observed, resulting in multidrug resistance in tumor cells through its transcriptional regulatory function.^[^ ^28^ ^]^ The 33 N‐terminal amino acids of APEX1 are reportedly essential for its biological functions, and APEX1‐WT, rather than APEX1‐NΔ33 (deletion of the 33 N‐terminal amino acids), induces cisplatin resistance via transcriptional regulation or the BER pathway.^[^ ^28^, ^29^ ^]^ Here APEX1 induced the expression multidrug resistance‐related genes and promoted PARPi resistance in ovarian cancer in response to PARPi treatment (**Figure** **7**A,B), indicating essential roles of APEX1 in PARPi resistance. However, APEX1‐NΔ33, which can bind both KAT6A and PARP1 (Figure S6G, Supporting Information), also enhanced PARPi resistance in SKOV3‐R cells (Figure 7B), demonstrating that APEX1 counteracts PARPi‐mediated cytotoxicity independent on its transcriptional regulatory function or the BER pathway. In PARP1‐Δ or KAT6A‐KO SKOV3‐R cells, the KAT6A‐PARP1‐APEX1 complex was disrupted, and APEX1‐WT or APEX1‐NΔ33 overexpression had no significant influence on PARPi resistance (Figure 7C–F; Figure S6H,I, Supporting Information).

To further study the molecular mechanisms by which APEX1 promotes PARPi resistance, we examined the effect of APEX1 on PARPi treatment‐induced PARP1 trapping. APEX1 knockdown enhances PARP1 trapping, and both APEX1‐WT and APEX1‐NΔ33 impair PARP1 trapping induced by PARPi treatment (Figure 7G). In contrast, the catalytic activity of PARP1 during PARPi treatment was analyzed by detecting PAR chains on XRCC1 or PARP1 (substrates of PARP1 as a poly‐ADP‐ribosyltransferase) using PLA assays. Regulation of APEX1 had no significant effect on the catalytic activity of PARP1 during PARPi treatment (Figure 7H,I). Furthermore, APEX1 knockdown had no significant influence on the amount of trapped PARP1‐Δ, which impairs the formation of KAT6A‐PARP1‐APEX1 complex, trapped at the DNA break sites (Figure 7J), indicating the essential role of the KAT6A‐PARP1‐APEX1 complex in PARP1 trapping.

Taken together, these results indicate that APEX1 impairs PARP1 trapping by enhancing KAT6A LLPS, and that the interaction between KAT6A and PARP1 is independent of its transcriptional regulatory function in ovarian cancer.

## 3 Discussion

Understanding and overcoming resistance to PARPis are the major research goals of many researchers. PARPis are an effective targeted therapeutic agent for HR‐deficient tumors that have been successfully applied in clinical practice. Recent findings have shown the curative effect of PARPi treatment in cancer depends mainly on its capacity to trap PARP1 on chromatin; thus, platinum‐sensitive ovarian cancer patients without HR‐deficient tumors could also benefit from PARPi treatment. However, most patients develop drug resistance.^[^ ^1^ ^]^ The best‐studied mechanisms of resistance to PARPi include drug target‐related resistance,^[^ ^5^, ^30^ ^]^ restoration of homologous recombination,^[^ ^31^ ^]^ restoration of replication fork stability,^[^ ^32^ ^]^ and removal of inhibitors from the cell by efflux transporters.^[^ ^33^ ^]^ Few studies have investigated the mechanism by which cancer cells escape the cytotoxic effects of PARP1 trapping.

In this study, we found that KAT6A levels were higher in patients with PARPi‐resistant ovarian cancer than in those with primary ovarian cancer. We conjectured that ovarian cancer cells with high KAT6A levels survive PARPi treatment and become the dominant clone, resulting in PARPi resistance, which was validated by in vitro experiments. In addition, KAT6A undergoes phase separation and adsorbs PARP1 into droplets independently of its catalytic function, decreasing the amount of PARP1 trapped within DNA break sites. APEX1 promotes multidrug resistance in cancer, enhances KAT6A LLPS, and facilitates interaction between KAT6A and PARP1. A stable complex containing KAT6A, PARP1, and APEX1 prevents PARP1 from binding to DNA lesions, thus impairing the cytotoxic effects of PARPi treatment.

KAT6A, a member of the MYST histone acetyltransferase family, is known to form a complex with EAF6, ING5, and BRPF1. It acetylates lysine residues of histone H3, thereby regulating multiple biological functions, including gene transcription, cell cycle, senescence, and signal transduction.^[^ ^11^ ^]^ Several studies have shown that the catalytic functions of KAT6A as an acetyltransferase are vital to the occurrence and development of ovarian cancer,^[^ ^6^ ^]^ leukemia,^[^ ^7a^ ^]^ breast cancer,^[^ ^34^ ^]^ and other tumors.^[^ ^11^ ^]^ However, in this study, histone acetylation in PARPi‐resistant ovarian cancer cells was not increased compared to that in parental cells, and the catalytic inhibition of KAT6A was incapable of restoring PARPi sensitivity, indicating that catalytic functions do not play key roles in resistance to PARPi in ovarian cancer. At present, studies on KAT6A have mainly focused on its acetyltransferase function; however, there are few reports on its non‐catalytic function. An increasing number of studies are exploring the noncatalytic functions of enzymes in addition to their catalytic activity.^[^ ^35^ ^]^ Here, we demonstrated that the destruction of KAT6A LLPS, rather than catalytic inhibition, increased PARP1 trapping and restored sensitivity to PARPi, providing potential therapeutic strategies for PARPi‐resistant ovarian cancer.

PARP1, the key target of PARPi, participates in DNA damage repair, a sophisticated process involving multiple factors. In this process, PARP1 synthesizes PAR chains both on substrate proteins (heteromodification) and itself (automodification), which initiates DNA repair by driving the recruitment/concentration of downstream DNA repair effectors and modulating chromatin structure. Auto‐PARylation promotes the release of PARP1 from damaged DNA, while chromatin retention of PARP1 by PARPi inhibits the recruitment and concentration of DNA repair effectors. Strikingly, the loss or diminished expression of PARP1 in tumor cells causes PARPi resistance,^[^ ^5^ ^]^ demonstrating the key role of PARP1 trapping in subsequent DDR and PARPi‐mediated cytotoxicity. Here, KAT6A LLPS also enhanced the subsequent DDR capacity by impairing PARP1 trapping and reducing PARP1‐DNA complexes, which attenuated the cytotoxic effects of PARP1 trapping, leading to resistance to PARP inhibitors in ovarian cancer cells. Targeting KAT6A LLPS or the interaction between KAT6A and PARP1 impairs DDR capacity and enhances the therapeutic effects of platinum‐based combination therapies.

LLPS, a reversible molecular process involving proteins or nucleic acids, participates in many biological activities, including gene transcription,^[^ ^36^ ^]^ genome organization,^[^ ^37^ ^]^ epigenetic modification,^[^ ^38^ ^]^ and signal transduction.^[^ ^39^ ^]^ Moreover, LLPS of cancer‐related proteins is important to the occurrence, development, and drug resistance of tumors.^[^ ^40^ ^]^ KAT6A can form aggregated droplets in the nuclei of PARPi‐resistant ovarian cancer cells, and KAT6A LLPS is essential for the interaction between KAT6A and PARP1. PARP1 proteins are recruited in droplets of KAT6A, which can be regarded as a “container” during PARPi treatment, decreasing the quantity of PARP1 proteins trapped in damaged DNA. Phase separation can store high concentrations of proteins in the form of droplets and release the proteins into the cell environment when the cell needs to regulate the concentration of relevant proteins in the cell.^[^ ^18^, ^41^ ^]^ In addition, LLPS can also mediate the localization of some proteins to phase separation droplets or membraneless organelles, or isolate proteins from their substrates.^[^ ^41^ ^]^

APEX1, which is essential for KAT6A LLPS, may act as a scaffold protein for the KAT6A‐PARP1‐APEX1 complex. Scaffold proteins promote the formation of stable complexes, which subsequently enhance LLPS.^[^ ^18^, ^42^ ^]^ Aberrant phase separation of KAT6A, assisted by APEX1, regulates the subcellular distribution of PARP1 and decreases PARP1 trapping‐mediated cytotoxic effects of PARPi treatment, leading to resistance to PARPi in ovarian cancer cells. The KAT6A test has broad prospects for predicting and tracking PARPi responses during treatment, which warrants further investigation. Targeting KAT6A reverses PARPi resistance, indicating a promising avenue for extending the clinical utility of PARPis.

Aberrant LLPS and the transition of certain molecules are causal factors in a variety of human diseases, and these findings provide new ideas for anticancer therapeutic strategies and a new concept for anticancer drug design.^[^ ^40^ ^]^ However, the precise conformational transition in the KAT6A LLPS process remains unknown due to inadequate knowledge and biotechnology. More studies are necessary to investigate which conformation of KAT6A binds more PARP1,^[^ ^43^ ^]^ as targeting a certain conformation rather than catalytic inhibition may result in better curative effects.

## 4 Conclusion

In summary, KAT6A LLPS promotes PARPi resistance in ovarian cancer independent of its catalytic activity. KAT6A impairs PARP1 trapping by forming a stable KAT6A‐PARP1‐APEX1 complex. KAT6A LLPS, facilitated by APEX1, inhibits the cytotoxic effects of PARP1 trapping and enhances DNA damage repair during PARPi treatment. Our study provides evidence that KAT6A LLPS may be a therapeutic target and an indicator of PARPi responsiveness.

## 5 Experimental Section

### Cell Lines

The SKOV3, A2780, and HEK293T cells used in this study were obtained from Cell Bank, Chinese Academy of Sciences. ID8 was purchased from Yan‐Sheng Biotechnology (Shanghai). UWB1.289 cells were kindly provided by Shanghai Whelab Bioscience Limited. All cell lines were cultured in medium following the manufacturer's protocol. All cells were confirmed to be mycoplasma‐free using a mycoplasma‐detecting test (Yeasen, Shanghai).

### Mice

Female Balb/c nude and WT C57BL/6 mice were purchased from Shanghai SLAC Laboratory Animal Co. Ltd (Shanghai, China). All animal experiments were carried out according to the guidance of the ethics committee of Fudan University.

### Patient Samples

Ovarian cancer tissues were obtained from patients diagnosed with ovarian cancer in the Department of Obstetrics and Gynecology, Shanghai General Hospital and Zhongshan Hospital, Fudan University. Informed written consent was obtained from all patients. The research was approved by the Ethics Committee at Shanghai General Hospital. Informed written consent was obtained from all patients.

### Antibodies and Reagents

The following antibodies were used in this study: anti‐PARP1 (9532), KAT6A (85 460), HA (3724), PALB2 (30 253), Acetylated Lysine (Ac‐Lys) (9441) (Cell Signaling Technology); anti‐γH2AX (phos‐Ser139) (ab81299), Rad51 (ab133534), PARP5a (ab227469), FUS (ab124923), BRCA1 (ab131360), APEX1 (ab189474), Histone H3 (acetyl K9) (ab32129), Histone H3 (acetyl K14) (ab52946), Histone H3 (acetyl K23) (ab177275), KAT6B (ab246879), PARP16 (ab154510), Histone H3 (ab1791) (Abcam); anti‐Flag M2 (F3165) (Sigma‐Aldrich); anti‐β‐actin (66009‐1‐Ig), GAPDH (60004‐1‐Ig) and β‐Tubulin (66095‐1‐Ig) (Proteintech Group); The secondary antibodies were from Life Technology or Cell Signaling Technology. Cell culture media and other reagents were from Invitrogen, Gibco, and Fisher Scientific. WM‐1119, olaparib, talazoparib, and cisplatin were purchased from MedChemExpress (MCE).

### Plasmids

KAT6A and PARP1 cDNAs were amplified from the total cDNA of HEK293T cells, pLVs‐blast‐FUS was purchased from the DNA core at Shanghai Jiao Tong University, and APEX1 cDNA was obtained from Saiheng Biotechnology (Shanghai). Then the cDNAs were subcloned into the pLVs‐blast (from DNA core in Shanghai Jiao Tong University), PB‐TRE3G (obtained by deleting SOX17 of PB‐TRE3G‐SOX17, Addgene #104 541) or PCR3.1 vector. Point mutations or truncations were generated using the Q5 Site‐Directed Mutagenesis Kit (NEB) following the manufacturer's protocol.

### Immunoblotting (IB) and Immunoprecipitation (IP) Assays

IB and IP assays were performed as described previously.^[^ ^44^ ^]^ In brief, cells or tissues were collected and lysed in RIPA buffer (20 mm Tris‐HCl, pH 7.5, 150 mm NaCl, 1 mm EDTA, 2 mm Na3VO4, 5 mm NaF, 1% Triton X‐100) with a protease inhibitor cocktail (Invitrogen). For immunoprecipitation assays, the protein lysates were incubated with the indicated antibodies, captured by protein G beads (Invitrogen), and eluted with 4x sample loading buffer. The proteins were resolved by SDS‒PAGE, transferred to PVDF membranes, and detected by the appropriate antibodies. The membranes were then incubated with the indicated secondary antibody and detected by an infrared imaging technique (Bio‐Rad Laboratories, Inc.).

### Colony Formation Assay

Five hundred ovarian cancer cells of the indicated genotypes were seeded in 6‐well plates. Cells were incubated for 14 days with or without the appropriate treatment, and colonies were fixed with methyl alcohol and stained with crystal violet. The number of colonies was counted and analyzed using Prism software (GraphPad). The assays were performed in triplicate.

### Immunohistochemistry

Ovarian cancer tissues were cut and fixed in 4% paraformaldehyde (PFA) for 24 h. Fixed tissues were then embedded in paraffin, sectioned, pretreated, and incubated with KAT6A antibodies. The tissue sections were treated with GTVisionTM III Detection System/Mo & Rb/including DAB (Gene Tech, Shanghai) followed by nuclear staining using hematoxylin (Sigma‒Aldrich). Images of the stained sections were obtained with a Leica Versa 8 system. IHC staining was scored as 0–7 according to the percentage of positive cells as reported previously.^[^ ^6^ ^]^ Two independent pathologists who were blinded to the slides examined and scored each sample as follows: 7, strong staining in ≈50% of tumor cells; 6, weak staining in ≈50% of tumor cells; 5, strong staining in ≈25% of tumor cells; 4, weak staining in ≈25% of tumor cells; 3, strong staining in ≈5 to 25% of tumor cells; 2, weak staining in ≈5‐‐25% of tumor cells; 1, low or no staining in < 1% of tumor cells; and 0, no detectable staining in any tumor cell (0%). Staining scores were analyzed using Prism software.

### Apoptosis Assay

Apoptosis assays were performed using an Annexin V Apoptosis Detection Kit according to the manufacturer's instructions. Cells were collected and washed 3 times. Cells were stained with Annexin V and propidium iodide (PI) for 15 mins. After 3 washes, the cells were analyzed using a BD FACSCanto flow cytometer (Becton Dickinson, Mountain View, CA, USA).

### sgRNA‐Mediated Gene Knockout, shRNA‐Mediated Gene Knockdown and Transfection

The lentiCRISPRv2 (Addgene #52 961) vector used for CRISPR–Cas9‐mediated gene knockout was obtained from Lab Feng Zhang. The sequences for the sgRNAs targeting KAT6A were CATACCACTGTTGCCACAGT and TTCGAGTGAAGGCCTTACGG. The sequences for the sgRNAs targeting KAT6B are TGAAAGACGGACCGCAGTAC and GTTGTCTGGGTCCTTATAGG. The sequences for the sgRNAs targeting APEX1 are GTAACGGGAATGCCGAAGCG and TTCACGCCACAAGAGCGCCA. All shRNA plasmids used in this study were purchased from the DNA core at Shanghai Jiao Tong University. Certain plasmids with packaging plasmids [pMD2. G (Addgene #12 259) and psPAX2 (Addgene #12 260)] were transfected into HEK293T cells using jetOPTIMUS (PolyPlus‐transfection) Transfection Reagent following the manufacturer's instructions. After 48 hours of transfection, virus‐containing supernatants were collected, concentrated, and transduced into appropriate cells.

### Chromatin Immunoprecipitation (ChIP)‐qPCR

ChIP assays were performed using ChIP Assay Kit (beyotime) following the manufacturer's instructions. Eluted DNA was measured by agarose gel electrophoresis and qRT‐PCR with input as a control. The data were analyzed by Prism software.

The following primers were used for ChIP‐qPCR analysis^[^ ^7^, ^45^ ^]^: MYC promoter‐F: AAGGGAGGCGAGGATGTGT; R: TTCGCCCTGGTTTTTCCAA; HOXA9 promoter‐F: GGGGAGACGGGAGAGTACAG; CGTCCAGCAGAACAATAACG.

### Measurement of Cellular Sensitivity to PARPi Treatment

2000 cells were seeded into 96‐well plates in 100 µl of medium per well, and the cells were continuously exposed to the appropriate concentrations of PARPi for 72 h in triplicate. Cell survival was determined by CELL TITER‐GLO assays (Promega) following the manufacturer's instructions. The ATP level in cells without treatment was defined as 100%. The percent viability of cells with the indicated treatment was defined as treated cells/untreated cells x 100.

### Metastasis Assay In Vivo

A total of 1 × 10^6^ ovarian cancer cells were injected into the abdominal cavity of mice as reported previously.^[^ ^6^, ^24^ ^]^ Treatment with PARPi (olaparib, 50 mg kg^−1^ d^−1^) and cisplatin (5 mg kg^−1^) was performed during week 2–4 post injection of cells. Mice with the indicated treatments were monitored and the survival status of the mice was recorded. All animal studies were approved by the Animal Care and Use Committee of Fudan University.

### Subcutaneous Xenograft Model

A total of 10^6^ cells resuspended in 100 µL PBS were injected subcutaneously into the right flanks of five‐week‐old female Balb/c athymic nude mice. Mice were treated with olaparib (50 mg kg^−1^ d^−1^) at weeks 2–4 post cell injection. After 4 weeks, the mice were humanely euthanized, and the tumor xenografts were removed, weighed, and collected for further research. All animal studies were approved by the Animal Care and Use Committee of Fudan University.

### Phase Separation Assay in Cells

SKOV3‐R cells expressing tagged KAT6A were seeded on coverslips. After adhering to coverslips, the cells were washed twice with PBS and fixed with 4% paraformaldehyde in PBS for 10 min. After two more washes in PBS, the cells were analyzed by confocal microscopy (Leica), and the visible puncta with diameters greater than 0.5 µm were defined as KAT6A puncta.

### FRAP Analysis

For the in vivo experiments, FRAP experiments were performed using a confocal microscope (Leica) with a 60× oil immersion objective. Defined regions were bleached with a 561‐nm or 488‐nm laser pulse (50% intensity, 0.5 s). The recovery fluorescence intensities from photobleaching were recorded for the indicated times. Analysis of the recovery curves was measured by the mean region of interest and further analyzed by Prism software.

### Mass Spectrometric Analyses

Proteomics analyses for Flag‐KAT6A or Flag‐KAT6A‐ΔIDR‐associated proteins were performed at PTM Biotech. Inc. (Hangzhou, China). Briefly, SKOV3‐R cells with indicated genotypes were collected and lysed. Then, the Flag‐KAT6A or Flag‐KAT6A‐ΔIDR‐associated proteins were isolated using anti‐Flag antibodies (SIGMA), and the complexes were accumulated by Protein G beads. The protein samples were analyzed by LC‐MS/MS using Q Exactive Plus (Thermo). The raw data were processed by MAXQUANT software. The raw data were searched against the UNIPROT database.

### Gene Ontology Analysis

Gene Ontology (GO) analysis compares gene sets of interest with functional annotations in the GO database to understand which functions or processes these genes are likely to be significantly related to. GO analysis was done through the “clusterProfiler” R package. The R package invokes the latest GO analysis database to ensure the timeliness of GO analysis.

### Chromatin Fractionation

The chromatin fractionation assay was performed using a subcellular protein fractionation kit (Thermo Fisher, 78 840) as reported previously.^[^ ^25^ ^]^ Briefly, cells with the indicated treatment were collected and washed with cold PBS. The cytosolic proteins were isolated after incubating the cells with CEB buffer for 10 min at 4 °C and centrifuged at 500 g for 5 min. The membrane proteins were isolated after incubating the previous pellet with the MEB buffer for 10 min at 4 °C and centrifuged at 3000 g for 5 min. The soluble nuclear proteins were isolated after incubating the previous pellet with NEB buffer for 30 min at 4 °C and centrifuged at 5000 g for 5 min. The chromatin‐bound proteins were isolated by incubating the previous pellet with the NEB buffer supplemented with nuclease for 15 min at room temperature and centrifuged at 16 000 g for 5 min. All procedures were performed following the manufacturer's instructions. Protein concentrations were determined by a BCA assay kit, and immunoblotting was carried out using standard procedures.

### Comet assays

Comet assays were performed as described previously.^[^ ^46^ ^]^ Briefly, cells were digested and collected after appropriate treatment. The cells were then processed for the alkaline comet assay using the Comet SCGE Assay kit (Enzo Life Sciences) following the manufacturer's protocol. The percentage of tail intensity represented the degree of DNA damage and was computed by Comet Assay IV software (Perceptive Instruments Ltd.).

### Immunofluorescence Assays

Cells were seeded on glass bottom cell culture dishes (NEST) and treated for the appropriate time. After treatment, the cells were fixed in 4% paraformaldehyde for 20 min, permeabilized with 0.5% Triton X‐100 in PBS for 20 min, and blocked with 10% goat serum in PBS for 2 h at room temperature. The cells were washed after incubation with appropriate antibodies overnight at 4 °C and incubated with secondary antibodies conjugated to Alexa 488 or Alexa 555. Nuclear was stained by DAPI. Images were obtained and analyzed by a confocal microscope system (Leica).

### Proximity Ligation Assays (PLA)

The PLA assays were performed using the Duolink In Situ Red Starter Mouse/Rabbit Kit (Sigma‒Aldrich) following the manufacturer's instructions. The primary antibodies used in this assay were: mouse anti‐PARP1 (Sigma‒Aldrich, WH0000142M1), rabbit anti‐PARP (Cell Signaling), rabbit anti‐KAT6A (Cell Signaling), mouse anti‐KAT6A (Abnova) and rabbit anti‐phospho‐H2AX (Cell Signaling). Images were acquired under a confocal microscope system (Leica). The PLA foci were counted and analyzed by Prism software.

### Quantitative Real‐Time RT PCR (qRT‒PCR) Assay

Total RNA was isolated from cells using the MolPure Cell/Tissue Total RNA Kit (YEASEN) and reverse‐transcribed using the 1st Strand cDNA Synthesis SuperMix Kit (YEASEN) according to the manufacturer's protocol. qRT**‒**PCR was performed using the DyNAmo HS SYBR Green qRT**‒**PCR kit (Finzymes) and a CFX Connect Real‐Time System (Bio‐Rad Laboratories). The relative expression level of the indicated genes was normalized against that of GAPDH.

### Statistical Analysis

The data were analyzed using Prism version 9.0 (GraphPad Software Inc.) and SPSS (IBM). P values were calculated using a paired or unpaired two‐tailed Student's *t* test for 2 groups and one‐way ANOVA for multiple groups. Survival analysis was calculated using the log‐rank test. The details of data presentation, sample size (n) for each statistical analysis, and statistical methods were demonstrated in the indicated figure legends. *P* values < 0.05 were considered to indicate statistical significance.

### Ethics Approval and Consent to Participate

All animal experiments were carried out according to the guidance of the ethics committee of Fudan University. (2022‐071). The samples of pathologically diagnosed ovarian cancer were collected from Shanghai General Hospital and Zhongshan Hospital, Fudan University, with written informed consent and approval from the Institutional Review Board–approved protocols (2023‐303).

## Conflict of Interest

The authors declare no conflict of interest.

## Author Contributions

Z.Z. and J.Z. contributed equally to this work. Z.Z., W.L., and L.H. conceived the study. Z.Z., J.Z., and W.L. performed the experiments. J.Z., H.L., and C.W. provided critical advice. Z.Z. and W.L. wrote the paper.

## Supporting information

Supporting Information

Supplemental Video 1

Supplemental Video 2

Supplemental Table S1

Supplemental Table S2

## Data Availability

The data that support the findings of this study are available from the corresponding author upon reasonable request.
