# Supplementary material for: KAT6A Condensates Impair PARP1 Trapping of PARP Inhibitors in Ovarian Cancer
Source: Adv Sci (Weinh). 2024 Jul 8;11(34):2400140. doi: 10.1002/advs.202400140 (PMC11425913; doi:10.1002/advs.202400140)
Supplement: Supplementary file 1 — Supporting Information [file ADVS-11-2400140-s004.docx]

SUPPORTING INFORMATION

KAT6A Condensates Impair PARP1 Trapping of PARP Inhibitors in Ovarian Cancer

*Zhiyan Zhan ^*^, Jiarong Zhang, Huisheng Liang, Chong Wang, Li Hong ^*^, Wenxue Liu ^*^*


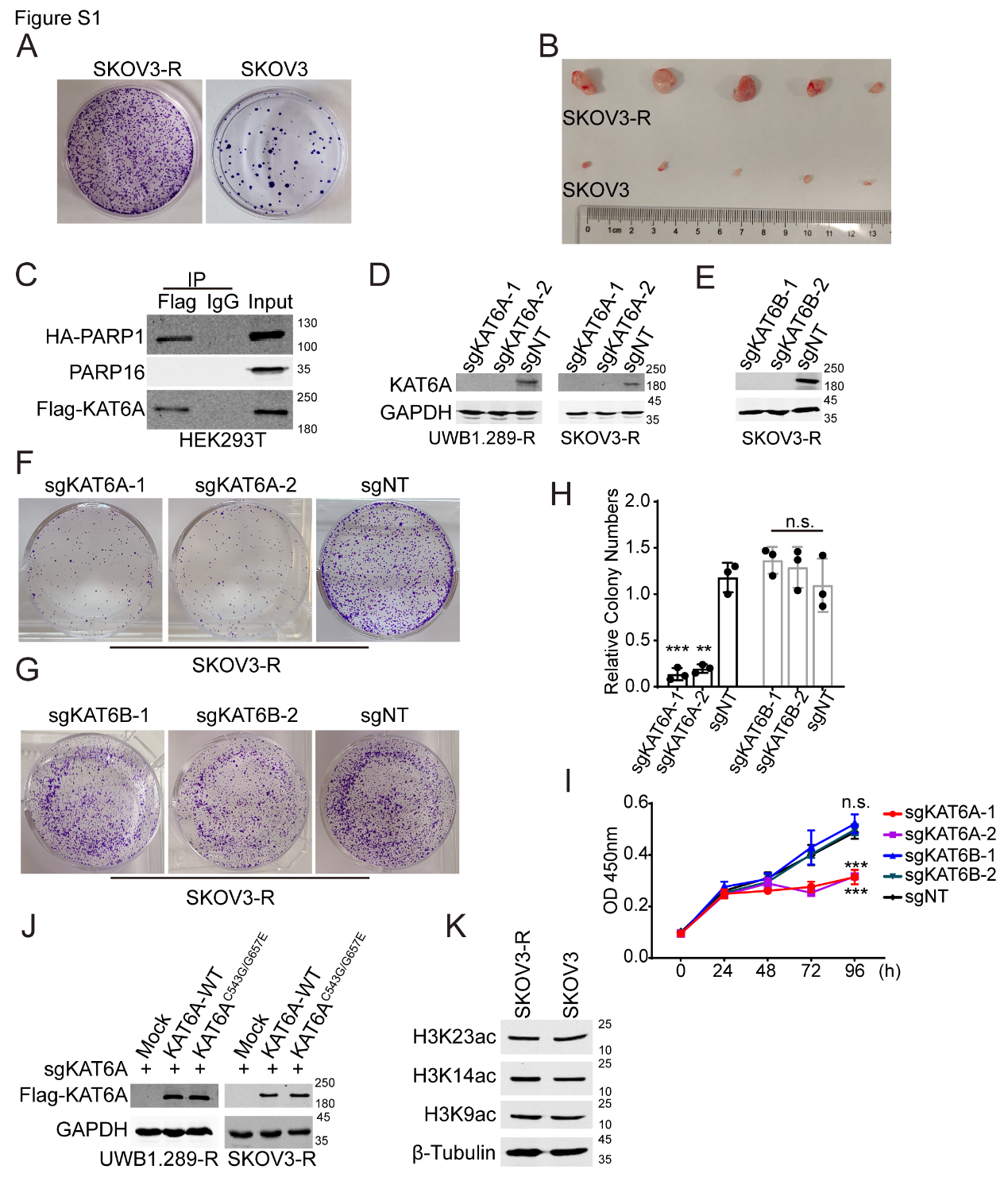


**Figure S1**

(A, B) PARPi resistance of SKOV3-R and UWB1.289-R cells was conformed using Colony formation assay (A, representative results from one of three independent experiments are shown) and subcutaneous xenograft model (B, n=5 per group). (C) The interaction of KAT6A and PARP1 was identified by Co-IP assays in HEK293T cells. PARP16 was set as negative control for Co-IP assays. (D, E) Knockout of KAT6A (D) or KAT6B (E) using CRISPR-Cas9 system in SKOV3-R and UWB1.289-R cells. (F-H) KAT6A knockout (F) but not KAT6B knockout (G) impairs PARPi resistance of SKOV3-R treated with olaparib. Representative results from one of three independent experiments are shown. Statistical analysis was shown in H (one-way ANOVA followed by Tukey’s multiple-comparison test, n=3 per group). (I) KAT6A knockout but not KAT6B knockout impairs PARPi resistance of SKOV3-R treated with olaparib. (J) Re-expression of KAT6A-WT or KAT6A^C543G/G657E^ in SKOV3-R and UWB1.289-R cells. (K) The levels of H3K9ac, H3K14ac and H3K23ac in PARPi-resistant or primary ovarian cancer cells. All data are expressed as mean ± SD. **P < 0.01; ***P <0.001; n.s. denotes no signification.


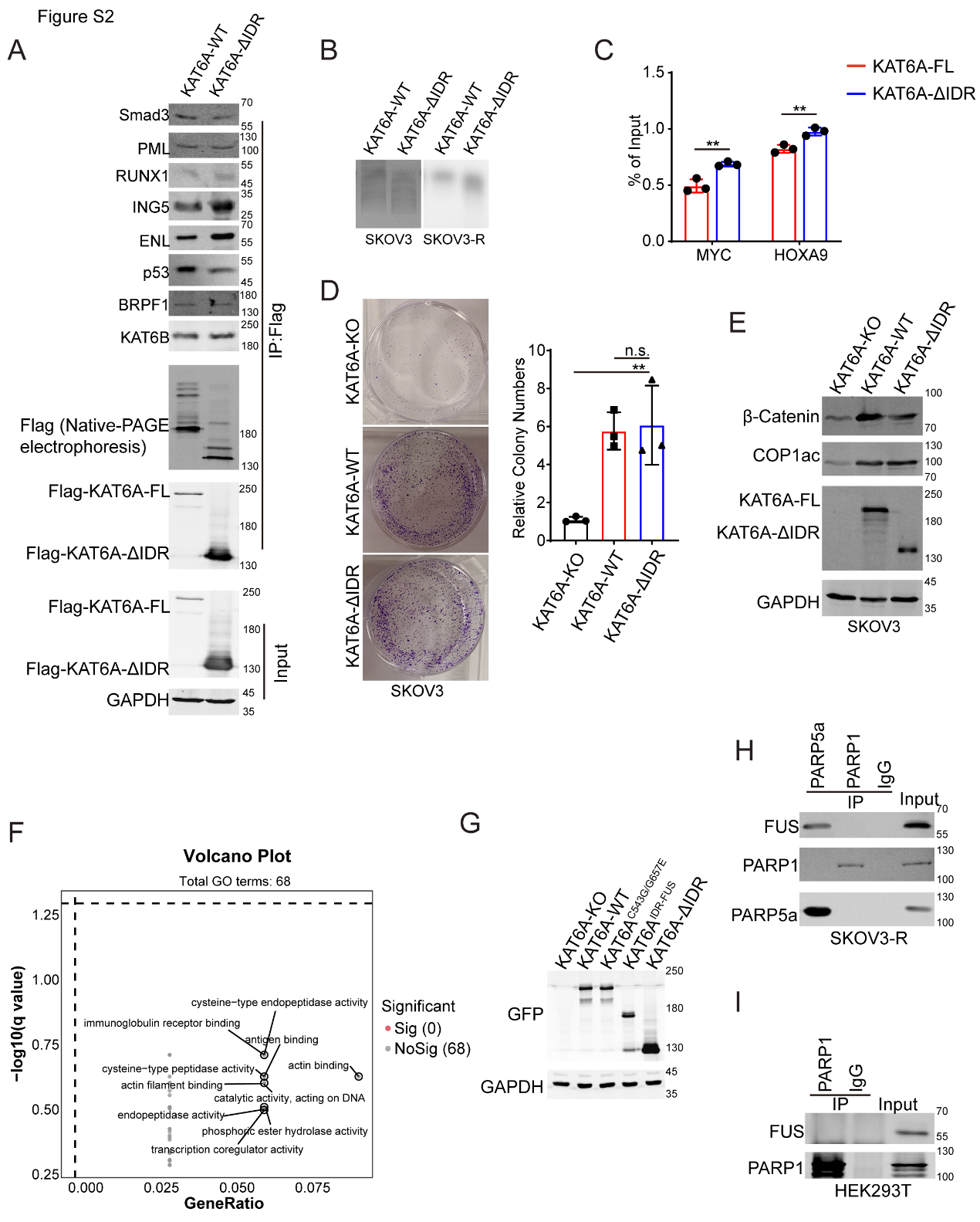


**Figure S2**

(A) Co-IP assays showed that KAT6A-ΔIDR can also bind indicated proteins that bind KAT6A-WT. And KAT6A-ΔIDR forms complex as KAT6A-WT does in Native-PAGE electrophoresis. (B, C) ChIP targeting KAT6A was performed and the eluted DNA were measured by agarose gel electrophoresis (B). ChIP-qPCR assays showed that deleting IDR domain doesn’t impair the ability of KAT6A to localizes to its hallmark regulatory loci, the HOXA9 and MYC promoter (C, Unpaired t-test, n=3 per group). Representative results from one of three independent experiments are shown. (D) Both KAT6A-WT and KAT6A-ΔIDR promote resistance of SKOV3 to cisplatin. The representative images are shown in left and statistical analysis was shown in right (one-way ANOVA followed by Tukey’s multiple-comparison test, n=3 per group). (E) Both KAT6A-WT and KAT6A-ΔIDR can acetylate COP1 and stable β-catenin in SKOV3. (F) GO analysis (Volcanic plot) of proteins that bind Flag-KAT6A-WT but not Flag-KAT6A-ΔIDR in mass spectrometry analysis following Co-IP targeting Flag. (G) The overexpression of GFP tagged KAT6A-WT, KAT6A^C543G/G657E^, KAT6A^IDR-FUS^, KAT6A-ΔIDR in SKOV3-R cells. (H, I) PARP1 doesn’t bind FUS in SKOV3-R cells (H) or HEK293T cells (I). PARP5a who was found to bind FUS was set as positive control. All data are expressed as mean ± SD. **P < 0.01; ***P <0.001; n.s. denotes no signification.


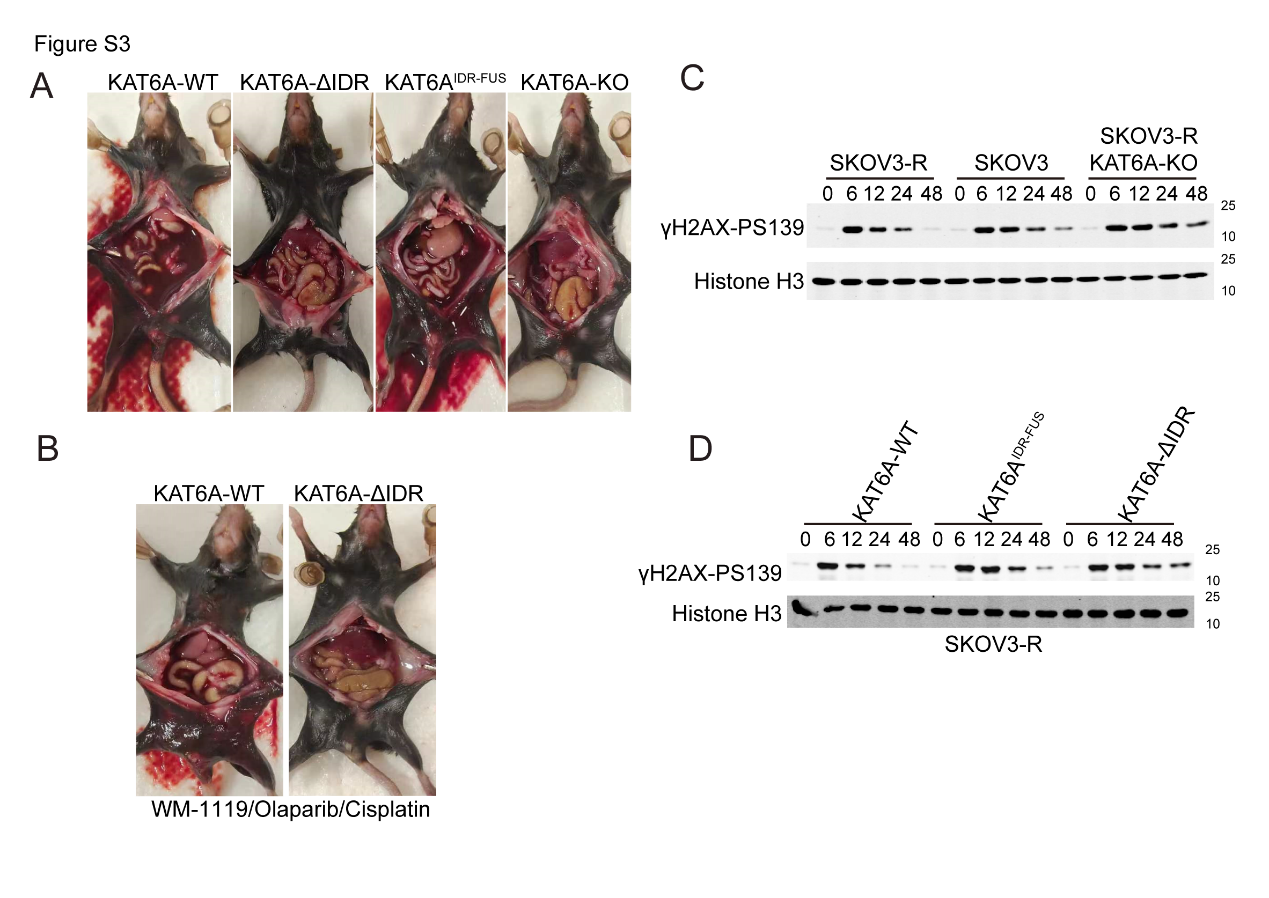


**Figure S3**

(A) Deleting IDR of KAT6A or KAT6A-KO of ID8-R cells reduced the ascites fluid formation of mice which induced by intraperitoneal implantation of ovarian cancer cells. Representative images from one of three independent experiments are shown. n=3 per group. (B) KAT6A-WT ID8-R cells induced more ascites fluid compared to KAT6A-ΔIDR ID8-R cells under WM-1119/olaparib/cisplatin treatment. Representative images from one of three independent experiments are shown. n=10 per group. (C) DDR was enhanced in SKOV3-R cells compared to that in SKOV3 cells. Enhanced DDR was impaired by KAT6A-KO in SKOV3-R cells. (D) Inhibiting LLPS of KAT6A by deleting IDR impairs the DDR in SKOV3-R cells. Rescuing LLPS of KAT6A enhances the DDR.


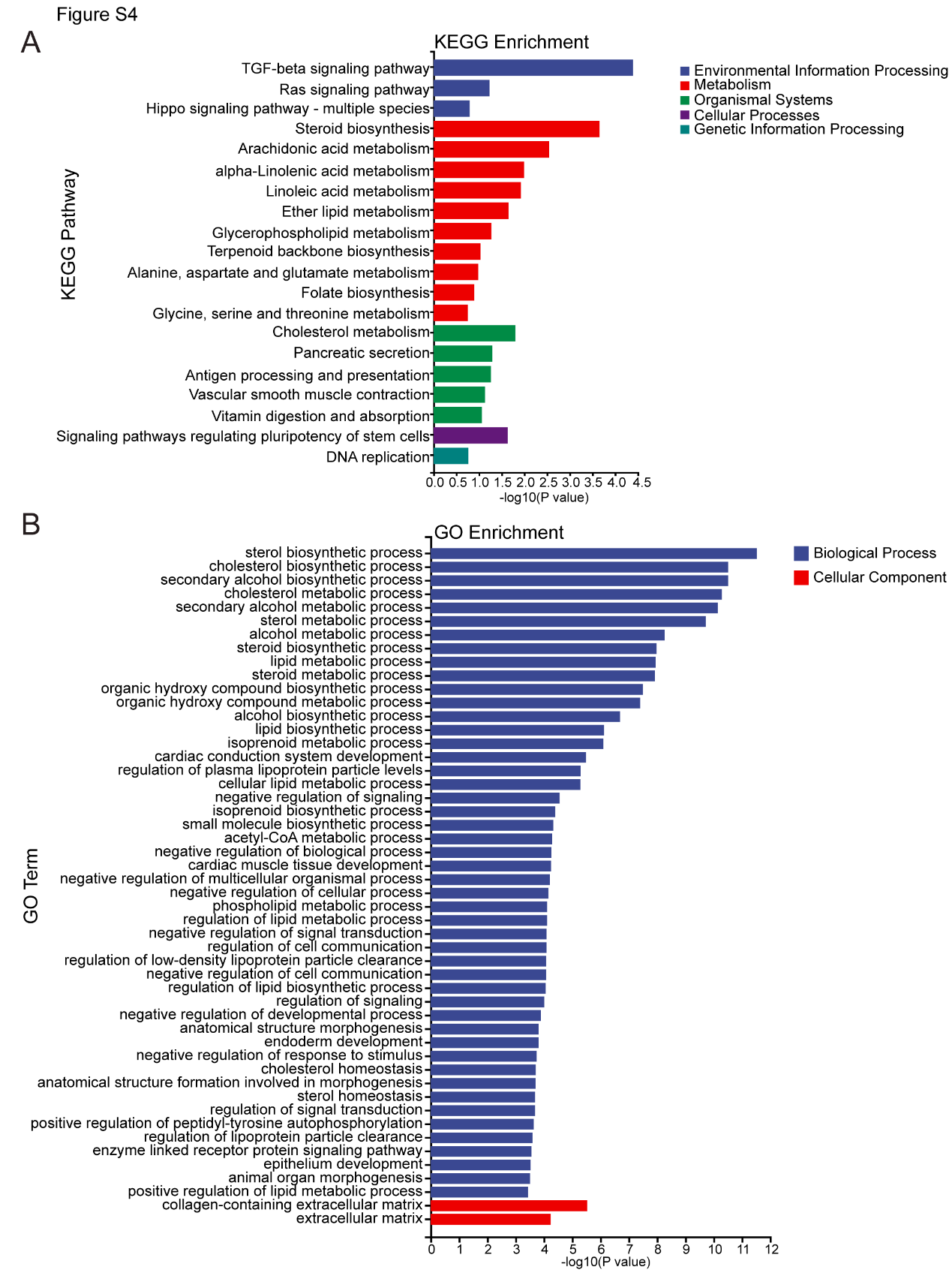
**Figure S4**

(A) KEGG analysis of genes differentiating between PARPi-resistant and parental SKOV3 cells by RNA sequencing. (B) GO analysis of genes differentiating between PARPi-resistant and parental SKOV3 cells by RNA sequencing.


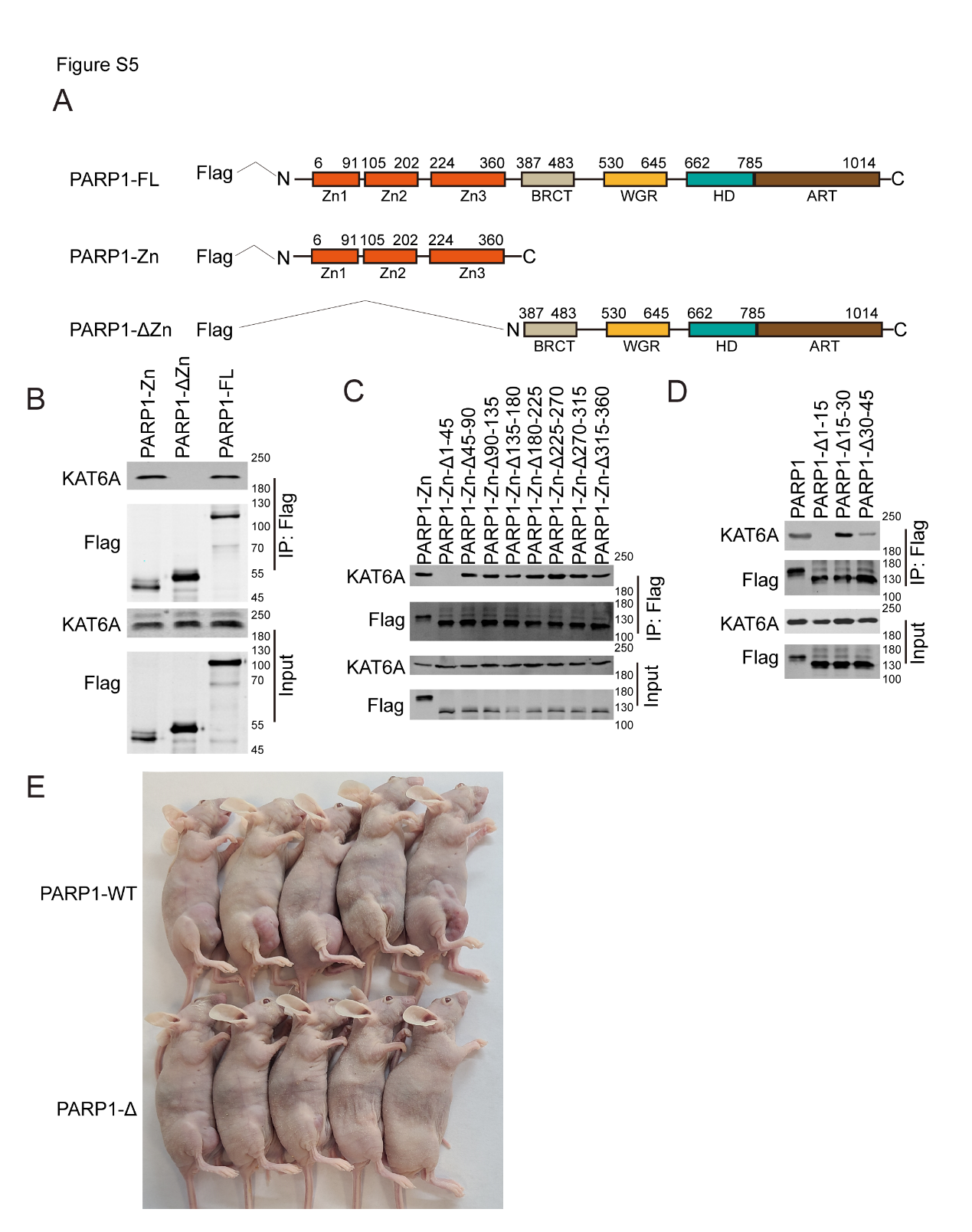


**Figure S5**

(A) The pattern diagram of full-length PARP1 (PARP1-FL) and truncated PARP1 protein. (B) Deleting Zn domain (PARP1-ΔZn) impairs interaction of PARP1 and KAT6A. (C) Deleting amino acid 1-45 impairs interaction of PARP1-Zn and KAT6A. (D) Deleting amino acid 1-15 impairs interaction of PARP1 and KAT6A. (E) The PARPi treatment inhibits PARP1-Δ SKOV3-R cells rather than PARP1-WT SKOV3-R cells. PARP1-Δ rescues the sensitivity of ovarian cancer to PARPi in vivo. Representative images from one of three independent experiments are shown. n=5 per group.


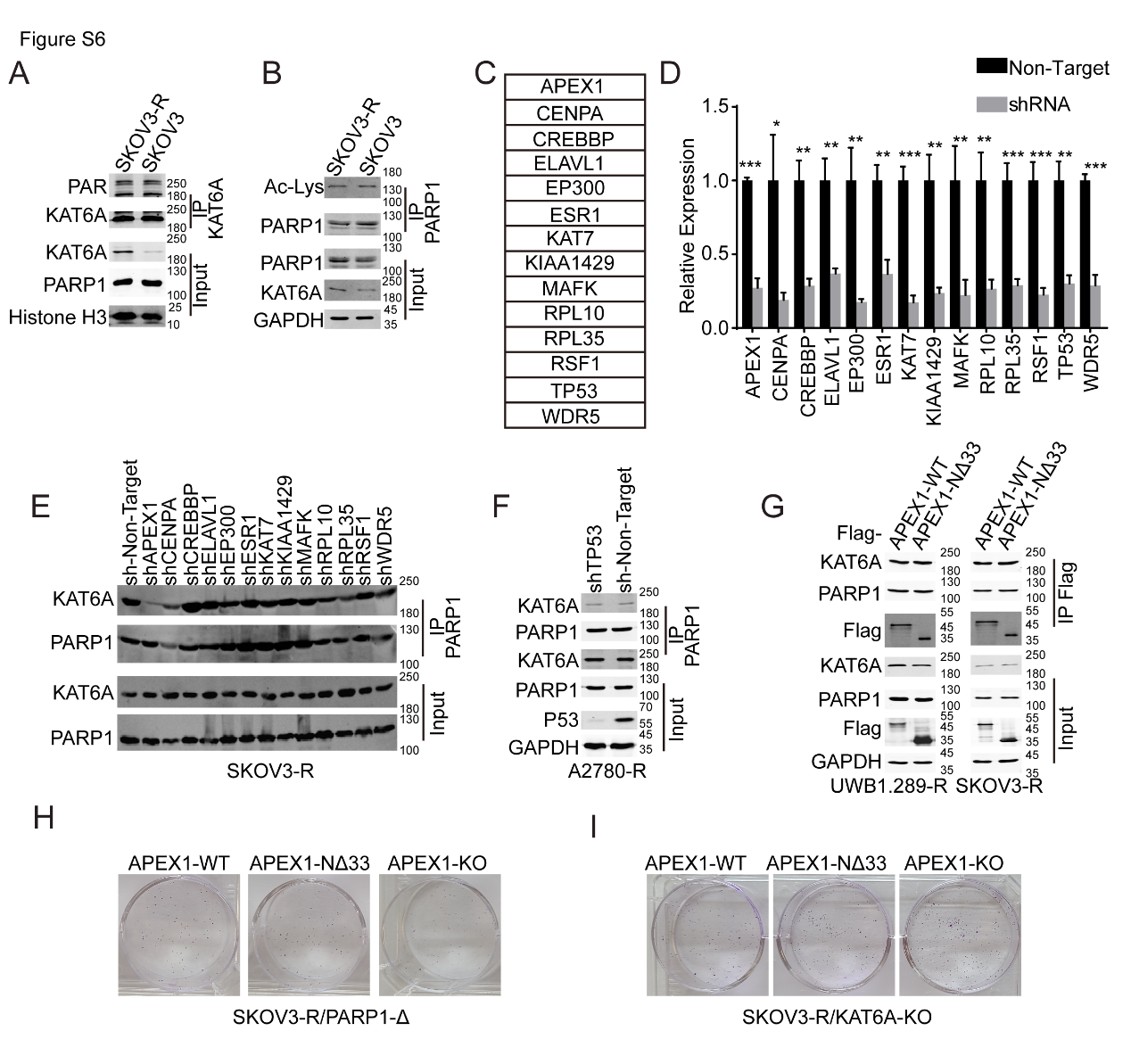
**Figure S6**

(A) No enhanced Poly (ADP-ribose) (PAR) of KAT6A was detected in PARPi-resistant SKOV3 cells compared to primary SKOV3 cells. (B) No enhanced lysine acetylation (Ac-Lys) of PARP1 was detected in PARPi-resistant SKOV-3 cells compared to primary SKOV-3 cells. (C) Selected 14 proteins that bind both KAT6A and PARP1. (D) Knockdown of indicated genes using shRNA in SKOV3-R cells (Unpaired t-test, n=3 per group). (E, F) The effects of inhibiting indicated genes on interaction of KAT6A and PARP1. WT-TP53 is expressed in A2780 but not in SKOV3 cells. (G) Both APEX1-WT and APEX1-NΔ33 bind KAT6A or PARP1. (H) APEX1-WT or APEX1-NΔ33 can’t rescue PARPi resistance in PARP1-Δ SKOV3-R cells. Representative images from one of three independent experiments are shown. (I) APEX1-WT or APEX1-NΔ33 can’t rescue PARPi resistance in KAT6A-KO SKOV3-R cells. Representative images from one of three independent experiments are shown. All data are expressed as mean ± SD. *P < 0.05; **P < 0.01; ***P <0.001

**Table S1**

KAT6A or KAT6A-ΔIDR-associted proteins.

**Table S2**

Proteins binding KAT6A but not KAT6A-ΔIDR.

**Video S1**

GFP-KAT6A condensates in SKOV3-R cell

**Video S2**

GFP-KAT6A-ΔIDR in SKOV3-R cell
